# Two-Step Approach to Obtain Nitrogen-Doped Reduced Graphene Oxide Using Nitrogen-Containing Reducing Agents

**DOI:** 10.1021/acsphyschemau.6c00049

**Published:** 2026-08-06

**Authors:** Carlos Belman-Rodriguez, Rodrigo Ponce-Pérez, Jose Ruiz-Marizcal, Gabriel Alonso-Nunez, Jose M. Romo-Herrera

**Affiliations:** † Centro de Nanociencias y Nanotecnología, Universidad Nacional Autónoma de México (CNyN-UNAM), Km. 107 Carretera Tijuana-Ensenada, Ensenada, Baja California 22860, México; ‡ Posgrado en Nanociencias, CICESE/CNyN-UNAM, Km 107 Carretera Tijuana-Ensenada, Ensenada, Baja California 22860, México

**Keywords:** reduced graphene oxide, nitrogen doping, N-containing reducing agents, dimethylformamide, ethylenediamine, wet chemistry

## Abstract

N-doping of graphene has emerged as a very valuable alternative for diverse applications. Nonetheless, further work is required to explore the grafting of N-containing molecules onto graphene to incorporate N atoms into its graphitic network, thereby harnessing a wide variety of available N-containing molecules. Here, the chemical reduction of graphene oxide (GO), using nitrogen-containing reducing agents such as ethylenediamine (EDA) and dimethylformamide (DMF), was explored to evaluate their effect on the surface composition of the obtained reduced graphene oxides (rGO). Afterward, an 800 °C thermal treatment was performed, and the samples were characterized before and after the thermal treatment, focusing on the nitrogen and oxygen functional groups present on the rGO. Several fingerprints were identified from the grafting of the EDA molecules functionalizing the rGO-EDA sample by XPS, FTIR, and UV–visible spectroscopy measurements and by first-principles calculations. The 800 °C thermal treatment led to significant changes in the surface chemistry, incorporating nitrogen as a dopant into the rGO to imprint the obtained N-rGO sample with electrocatalytic properties (Oxygen Reduction Reaction activity). Therefore, the proposed approach opens the possibility to explore a myriad of N-containing molecules for N-doping of rGO.

## Graphical abstract

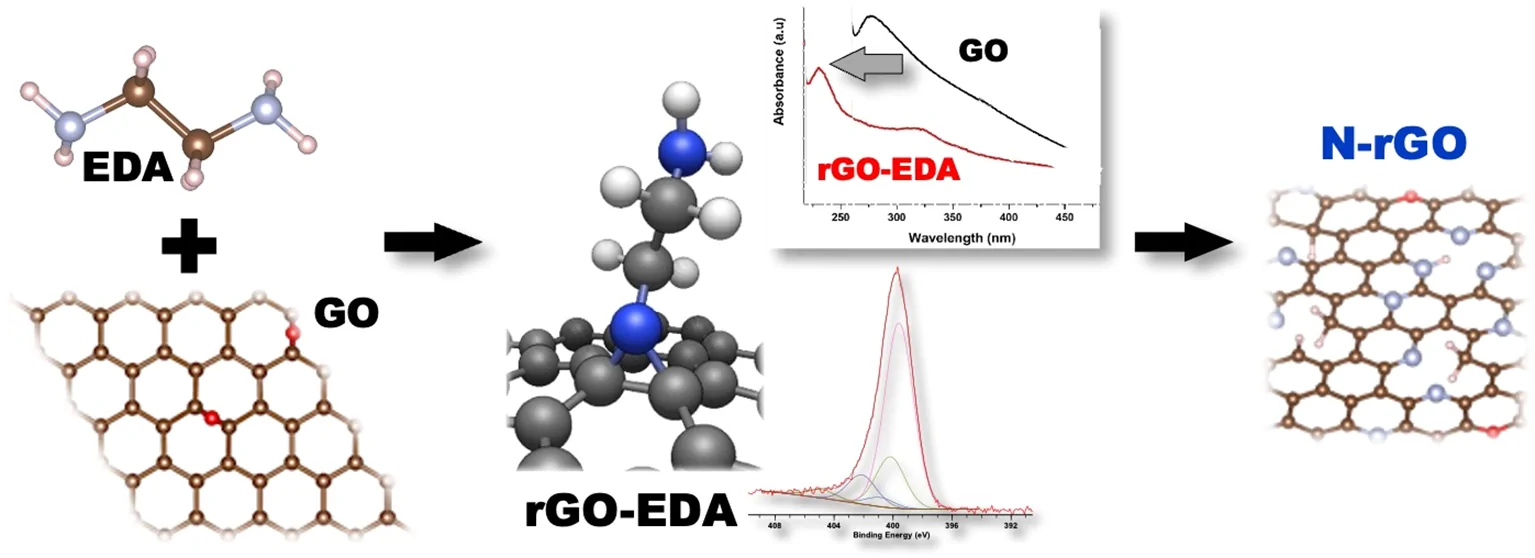

## 1 Introduction

N-doping of graphene has emerged^1–5^ as a very valuable alternative in several fields, including energy conversion and storage, with electrocatalysts for key reactions like the oxygen reduction reaction (ORR),^6–8^ oxygen evolution reaction (OER),^8–10^ and hydrogen evolution reaction (HER),^11,12^ as well as improved electrodes for supercapacitors (SCs).^13,14^ It has also presented options as electrocatalysts to convert CO_2_ into value-added chemical products such as ethanol,^15^ or to produce alternative fuels such as ammonia from nitrogen gas.^16^ Beyond electrochemical applications, N-doped graphene has shown good performance as sensors for gas, chemical, and biomolecule analytes;^17^ as adsorbents for pollutant removal, contributing to the environmental remediation;^18^ and in improving biocompatibility of carbon-based nanostructures for biomedical applications.^19^ N-doped graphene is becoming a remarkable nanostructured platform due to the possibility of tuning its electronic structure, presence of active sites, good chemical stability, great electric conductivity, robust mechanical properties, and high surface area.^2,20^ Moreover, the use of earth-abundant precursors makes this material highly accessible.^2,20^

Among the synthetic approaches to N-doped graphene, graphene oxide (GO) obtained by the oxidation of graphite has emerged as a particularly suitable precursor, because of its excellent dispersibility in aqueous solutions and the ease with which it can be chemically modified.^21^ Reducing GO to rGO (reduced graphene oxide) is crucial not only to restore the electrical and thermal conductivity, but also to be a key stage for incorporating nitrogen atoms into the carbon network. Various methods have been employed to convert GO into rGO, including thermal reduction at elevated temperatures^22,23^ or chemical reduction in aqueous media.^24,25^ In chemical reduction methodologies,^25–28^ reducing agents such as hydrazine, aluminum, vitamin C, and NaBH_4_ have been extensively employed. Among these, NaBH_4_ is considered to be one of the most efficient reducing agents. However, several nitrogen-containing molecules with reducing agent properties could also be explored.

The properties of rGO can be upgraded when doped with nitrogen, due to the nitrogen electronegativity, which causes a charge redistribution with its neighbor carbon atoms, inducing local “active regions” on the graphene surface.^13^ These active regions improve the materiaĺ’s ability to adsorb molecules and/or catalyze redox reactions. Moreover, the catalytic activity of this N-doped rGO has highly benefited from the induction of these active regions, ranging from electrocatalysts for the ORR^6–8,29^ to sequential electrochemical and chemical reactions by using intermediates from the electrochemical processes as reactants for subsequent chemical transformations.^4^ Specifically, the selectivity of the ORR toward the four-electron pathway or the two-electron pathway has been shown to directly depend on the type of nitrogen species (pyridinic N, pyrrolic N, or graphitic N) most abundant in the N-doped samples.^29–31^ The improvement in the ORR two-electron pathway has also opened the possibility of generating H_2_O_2_ on-site for use as a new reactant in subsequent chemical reactions to transform, for example, ethylene into ethylene glycol^32,33^ as a highly valuable chemical commodity. Nonetheless, further work is still required for exploring the grafting of N-containing molecules to functionalize graphene to later incorporate the nitrogen atoms into its graphitic network, opening the possibility to harness a myriad of N-containing molecules available to improve the development of scalable methods.

In this study, we explore the synthesis of nitrogen-doped rGO (N-rGO) from GO using nitrogen-containing reducing agents, such as dimethylformamide (DMF) and ethylenediamine (EDA), with a double role: as reducing agents and as grafting agents of nitrogen groups into the carbon network. The properties of the rGO obtained from these molecules were compared with those of a third rGO synthesized using NaBH_4_ as a control sample. In the initial stage, the reduction process was carried out at 80 °C to provide the thermal energy required to graft the N-containing molecules onto the rGO simultaneously during the partial removal of oxygen. When using EDA, the obtained rGO exhibited successful anchoring of the molecules by one of the amine groups on the carbon sheets. Experimental evidence is shown together with Density Functional Theory (DFT) calculations to assign these anchoring events and identify their fingerprints, including UV–visible, FTIR, and XPS spectra distinct signatures confirmed by first-principles calculations, revealing a favorable interaction between the epoxy and hydroxyl functional groups and resulting in covalent bonds to the rGO. As a second step, a thermal annealing treatment was performed at 800 °C, resulting in N-rGO due to structural reconfiguration. Finally, as a proof-of-concept, some electrochemical measurements are presented for the ORR activity of the different samples obtained.

Altogether, the evidence presented supports the feasibility of a two-step methodology for N-doping to obtain N-rGO. This approach enables the utilization of different N-containing molecules that play dual roles as reducing and N-doping agents. Moreover, the results highlight the importance of deepening the initially counterintuitive experimental signals, which unveil the characteristic fingerprint of the efficient anchoring of EDA molecules by their amine group into the rGO. Remarkably, the two-step N-doping approach proposed here opens the possibility to explore a myriad of N-containing molecules for the N-doping process of rGO to be harnessed by several fields making use of its characteristic properties, including energy-related applications.

## 2 Methods

### 2.1 Materials

Graphite flakes were obtained from Sigma-Aldrich and used as received. The oxidation process was carried out using H_2_SO_4_ (Sigma-Aldrich, 95–98%) and H_3_PO_4_ (Sigma-Aldrich) as the acidic reaction media, where phosphoric acid contributed to obtaining a less structurally damaged GO product, while KMnO_4_ (Jalmek) was employed as the oxidizing agent. Afterward, H_2_O_2_ (Jalmek, 30%) was added to terminate the oxidation reaction and eliminate insoluble manganese byproducts, whereas HCl (Sigma-Aldrich) was used during the washing process to remove residual metal species and impurities. NaBH_4_ (Sigma-Aldrich, 99.99%), ethylenediamine (EDA, C_2_H_4_(NH_2_)_2_, Sigma-Aldrich, 99.5%), and dimethylformamide (DMF, (CH_3_)_2_NCHO, Sigma-Aldrich, anhydrous 99.8%) were employed as reducing agents during the reduction of graphene oxide.

### 2.2 Synthesis Conditions

#### 2.2.1 Graphene Oxide (GO)

GO was synthesized using the method described by Marcano et al.^21^ To summarize, a mixture of H_2_SO_4_ and H_3_PO_4_ (9:1 vol:vol) was prepared with 252 mL of concentrated H_2_SO_4_ and 28 mL of H_3_PO_4_. The acid mixture was stirred, and subsequently, 12 g of KMnO_4_ was slowly dusted in 12 portions of 1 g each and afterward 2 g of graphite, taking care to avoid temperature increments exceeding 35–40 °C due to exothermic reactions. Afterward, the mixture was placed in a water bath at 50 °C and stirred for 12 h. Then, the mixture was removed from the water bath and allowed to cool to room temperature. Next, 280 mL of ice in deionized water and 4 mL of H_2_O_2_ were placed in a laboratory crystallizer. Then, the obtained mixture was gently poured into a laboratory crystallizer on ice under gentle stirring. Once all the mixture was well homogenized with the melted ice and H_2_O_2_ present in the crystallizer, the new mixture was transferred into reinforced centrifuge tubes (high-speed centrifuge tubes or Oak Ridge tubes). The mixture was centrifuged at 5000 rpm for 4 h to discard the supernatant. This was followed by four washing cycles, each consisting of centrifugation at 5000 rpm for 4 h using deionized water and HCl, and twice in ethanol. The solids collected were then redispersed in 200 mL of diethyl ether, filtered, and left to dry.

#### 2.2.2 Synthesis of Reduced Graphene Oxide (rGO)

The reduction of graphene oxide was achieved using three different reducing agents: sodium borohydride (NaBH_4_), ethylenediamine (EDA), and dimethylformamide (DMF).

(a) rGO from NaBH_4_.
  For each milligram of GO, 1 mL of deionized water was used to disperse it, and the pH of the solution was adjusted between 9 and 10 with a 5% NaOH solution. For every 20 mg of dispersed GO, 151 mg of NaBH_4_ was added. The mixture was stirred and heated in an oil bath at 80 °C for 2 h. Finally, the mixture was washed three times with deionized water by centrifugation at 13,000 rpm for 30 min to obtain the sample rGO-NaBH_4_.

(b) rGO from ethylenediamine (EDA).
  A total of 100 mg of GO was dispersed in 17 mL of water. Separately, a solution of 1.33 mL of EDA in 18 mL of ethanol was prepared. The EDA–ethanol mixture was added dropwise to the GO dispersion. The resulting mixture was stirred in an oil bath at 80 °C for 24 h. After this period, the mixture was washed three times by centrifugation at 13,000 rpm for 30 min to obtain the rGO-EDA sample.

(c) rGO from dimethylformamide (DMF).
  A total of 100 mg of GO was suspended in 34 mL of deionized water, and the mixture was stirred for 2 h to obtain a homogeneous dispersion. Subsequently, 3 mL of DMF was added to each mL of water in the dispersion. The mixture was then stirred and heated in an oil bath at 80 °C for 24 h. Finally, the mixture was washed eight times by centrifugation at 13,000 rpm for 30 min to obtain the rGO-DMF sample.

#### 2.2.3 Heat Treatments

After each reduction process, the three samples were thermally treated for 3 h at 800 °C in an inert atmosphere. The resulting materials were designated as rGO-NaBH_4__TT800, rGO-EDA_TT800, and rGO-DMF TT800.

### 2.3 FTIR Spectroscopy

Infrared spectra in the stretching vibration range from 600 to 1800 cm^–1^ were recorded on a Thermo Nicolet spectrophotometer with a resolution of 4 cm^–1^. Spectra were collected after 100 scans using KBr as the background.

### 2.4 X-ray Photoelectron Spectroscopy (XPS)

XPS spectra were acquired using a Scanning XPS microprobe PHI 5000 VersaProbe II system. The material was deposited directly onto the carbon tape to completely cover the tape area, mounted on a molybdenum sample holder, and placed in an ultrahigh vacuum (UHV) chamber for 12 h to remove solvent residues (water or volatile organics), achieving a vacuum of 7 × 10^–10^ Torr. Spectra were collected at a pressure of 1 × 10^–9^ Torr using a monochromatic Al Kα X-ray source (1486.6 eV) with a 200 um beam diameter and a Multi-Channel Detector (MCD) analyzer. Spectra were recorded at a 45° angle to the surface normal, under a pressure of 5 × 10^–7^ Pa, and with Constant Analyzing Energy (CAE) of 117.40 and 11.75 eV for the survey and high-resolution narrow scan modes, respectively. The surface samples were not etched, and the peak positions were referenced to the sp^2^ (C=C) carbon peak at 284.8 eV.^21,34–36^

The spectra were processed using Shirley’s reference correction for C and O high-resolution windows and Touggard’s correction for N. The deconvolution employed a GL(30) line shape with fwhm values of (0.8 to 3.0) for N, (1.0 to 3.0) for C, and (1.0 to 1.6) for O. The high-resolution C 1s peak was deconvoluted into species with the following binding energies: C–C (sp^2^) (284.8 – 284.8 eV), C–C (sp^3^) (285.9 – 285.5 eV), CO groups (hydroxyl, epoxide, ethers) (287.0 – 286.1 eV), C=O (carbonyls, ketones, aldehydes) (288.0 – 287.1 eV), O–C = O (carboxyl) (289.2 – 288.0 eV), and π–π* (291.0 eV), as noted in previous works.^21,34–36^ The high-resolution N 1s peak was deconvoluted with the following binding energies for various nitrogen species: pyridinic N (Npyr, 397.9–398.7 eV),^37–42^ pyrrolic N (Npyrrol 399.5–400.3 eV),^37,38,40–42^ graphitic N (Ngr. 400.9–401.7),^39–42^ oxidized N (402.0–403.5 eV),^31,41,43^ and molecular N (N_2_ 404.3–405.7 eV).^42–44^ It is worth noting the presence of a component assigned to molecular N_2_. Even if detected in trace amounts, they could be mechanically intercalated or trapped between graphene sheets or inside structural wrinkles resulting from doping processes. The high-resolution O 1s peak was deconvoluted using the following binding energies: O=C (quinones/carbonyls) (530.7–531.1 eV), anhydrides or lactones (C=O) and ether (O–C–O) (531.8–532.3 eV), ethers O in lactones or anhydrides (C–O–C) (532.8–533.3 eV), hydroxyl/carboxyl/phenolic (H–O–C) (533.7–534.2 eV), and adsorbed or intercalated water (H–O–H) [534.5–535.5 eV].^45–47^

### 2.5 Electrochemical Measurements

The obtained rGOs were dispersed in deionized water using a sonication bath. The resulting dispersion had a concentration of 10 mg/mL. By drop-casting, 0.30 mg of each of the previously dispersed samples was deposited onto the glassy carbon electrode (3 mm diameter, geometric area = 0.071 cm^2^), resulting in a catalyst loading of approximately 4.2 mg cm^–2^. Electrochemical measurements were carried out in a three-electrode cell containing a 0.1 M KOH electrolyte saturated with O_2_. A modified glassy carbon electrode was used as the working electrode, a platinum wire as the counter electrode, and Ag/AgCl (3 M KCl) as the reference electrode. All measurements were performed using an Autolab potentiostat PGSTAT302N. Cyclic voltammetry (CV) measurements were conducted at a scan rate of 20 mV s^–1^. Linear sweep voltammetry (LSV) was performed at rotation rates of 0, 100, 400, 900, 2500, and 3600 rpm. The LSV curves presented in Figure 7 correspond to measurements acquired under O_2_-saturated conditions and were/were not background-corrected using measurements collected under N_2_-saturated conditions.

### 2.6 Computational Methodology

Using computational modeling based on DFT, the adsorption of EDA and DMF on different GO samples was investigated. Calculations were done in the Vienna *Ab initio* Simulation Package (VASP) code.^48–51^ The exchange-correlation energy was treated according to the generalized gradient approximation (GGA) with Perdew–Burke–Ernzerhof (PBE) parametrization.^52^ The electron–ion interactions were simulated employing the Projected Augmented Waves (PAW) pseudopotentials^53,54^ with 500 eV as the energy cutoff. van der Waals interactions are considered in the calculation by the DFT-D3 method of Grime with zero-damping function.^55^ To simulate graphene oxide, the supercell method was employed. Each supercell is formed by a GO monolayer in a 5 × 5 periodicity with a vacuum space larger than 15 Å to avoid interactions between periodic slabs. In geometry optimization, convergence is achieved when force components and energy differences are less than 0.01 eV/ Å and 1 × 10^–4^ eV, respectively. The Brillouin zone was sampled using a 2 × 2 × 1 *k*-point grid according to the Monkhorst–Pack scheme.^56^ Noncovalent interactions were investigated employing the Critic2 software.^57,58^

Covalent bond formation was investigated using NEB calculations^59,60^ with five intermediate images. Ab initio molecular dynamics (AIMD) calculations were used to investigate the thermal stability of nitrogen-doped graphene during heat treatment. AIMD simulations were carried out in the NVT ensemble using the Nose–Hoover thermostat at 800 °C, with a step value of 1 fs for a total AIMD simulation time of 5 ps.

## 3 Results and Discussion

The reduction kinetics of GO induced by nitrogen-containing reducing agents (EDA and DMF) were initially monitored by UV–Visible spectroscopy and compared with the reduction obtained using NaBH_4_ as a reference model. As shown in Figure 1, the pristine GO samples (black curves) have a main peak around 230 nm, corresponding to the π → π* electronic transition of the C=C bond in the aromatic rings and a slight shoulder around 300 nm of the n → π* transition of the C=O bonds.^61^

**Figure 1:**
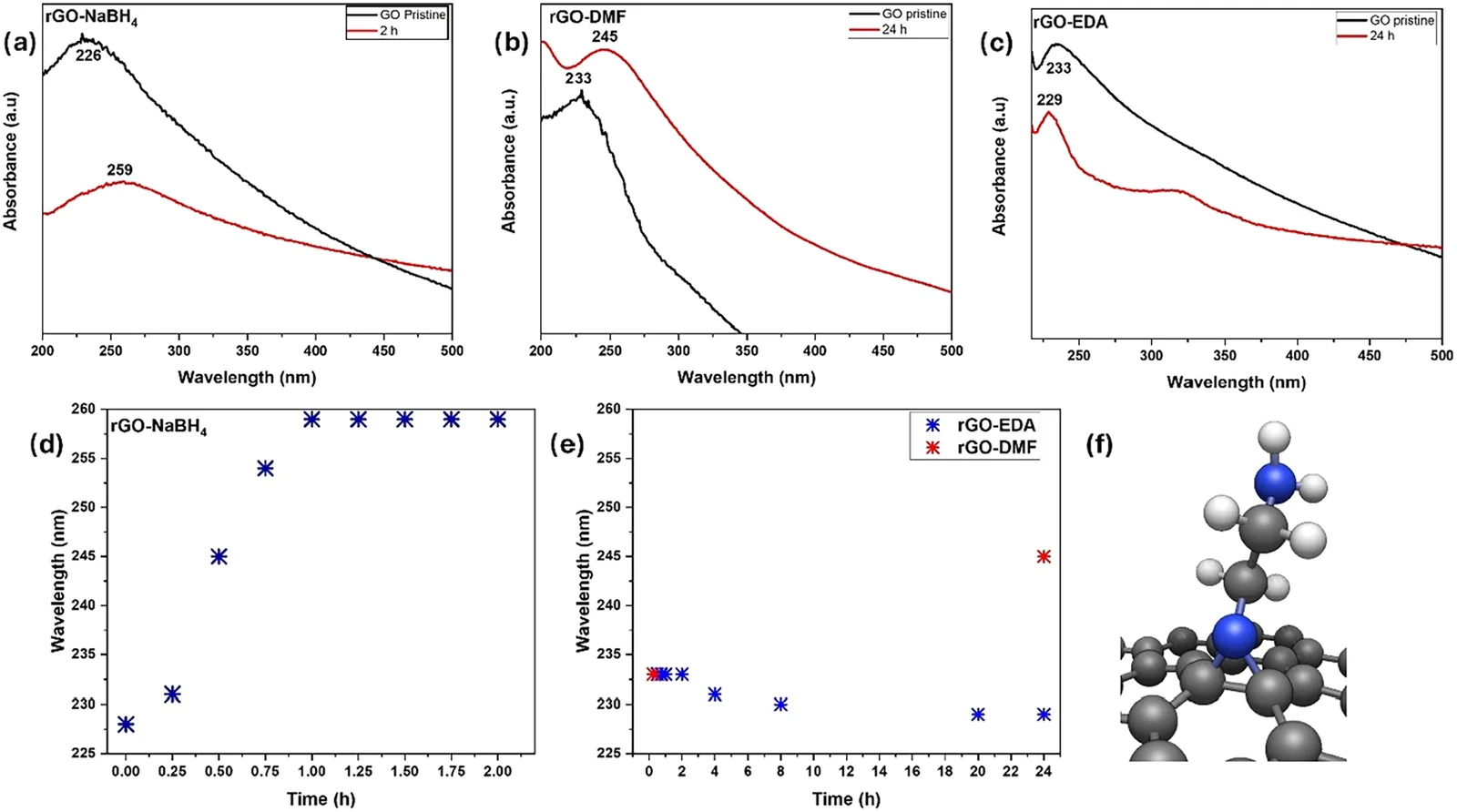
(a–c) Absorbance graphs obtained by UV–visible spectroscopy for rGO-NaBH_4_, rGO-EDA, and rGO-DMF. (d, e) Reduction kinetics showing the wavelength (nm) of the absorbance peak vs time (h) with different reducing agents. (f) Schematic drawing of an initial assumption of EDA molecule anchoring when used as the reducing agent.

The sample reduced with NaBH_4_ used as the reference shows a typical behavior with the absorbance band shifting toward longer wavelengths.^62^ The band shifts from 226 to 259 nm (Figure 1a). Similarly, rGO-DMF (Figure 1b) starts displaying the absorbance peak initially centered at ∼233 nm and shifts to longer wavelengths, but to a lesser extent (∼245 nm). This behavior indicates the restoration of the conjugated structure of the graphene sheets following the removal of oxygen-containing functional groups.

Unexpectedly, rGO reduced with EDA (Figure 1c) exhibits contrasting behavior. In this case, the absorbance band shifts toward shorter wavelengths, from 233 to 229 nm. To evaluate the reduction reaction kinetics for GO with different reducing agents, aliquots were taken at different time intervals. The evolution of the reduction process is depicted in Figure 1d,e, showing the absorbance band shift as a function of reaction time. For rGO-NaBH_4_, the maximum reduction degree was achieved within the first hour, indicating a fast and efficient reaction. In this case, aliquots were collected every 15 min over a 2 h period. Samples reduced with DMF and EDA (rGO-DMF and rGO-EDA) exhibited longer reaction times (Figure 1e), taking up to 24 h to complete. The rGO-DMF sample presented a significantly shorter shift when compared to rGO-NaBH_4_, resulting in ∼245 nm after 24 h of reduction time. For the case of rGO-DMF (Figure 1e), only the data corresponding to the initial condition (0 h) and the final condition (24 h) are shown to highlight the red shift for this sample, similar to the behavior observed for rGO-NaBH_4_. Strikingly, the rGO-EDA sample showed a blue shift in absorbance, beginning to appear after 4 h of reaction and completing after 20 h. This blue shift could be associated with the electron-withdrawing effect of some functional groups being attached, which could be consistent with the covalent bonding of EDA molecules to the graphene sheets, specifically at the epoxy group sites during the reduction process by the substitution of oxygen atoms with the nitrogen from the EDA molecule to enable its anchoring. Figure 1f illustrates a schematic drawing of this ansatz or the initial assumption. However, further evidence needs to be explored in later sections.

To gain further insight into the chemical composition of the rGO samples after reduction processes, with special emphasis on their nitrogen content, the reduced samples were characterized by FTIR and XPS. Figure 2 shows the characterization results of the rGO samples obtained using FTIR and XPS.

**Figure 2:**
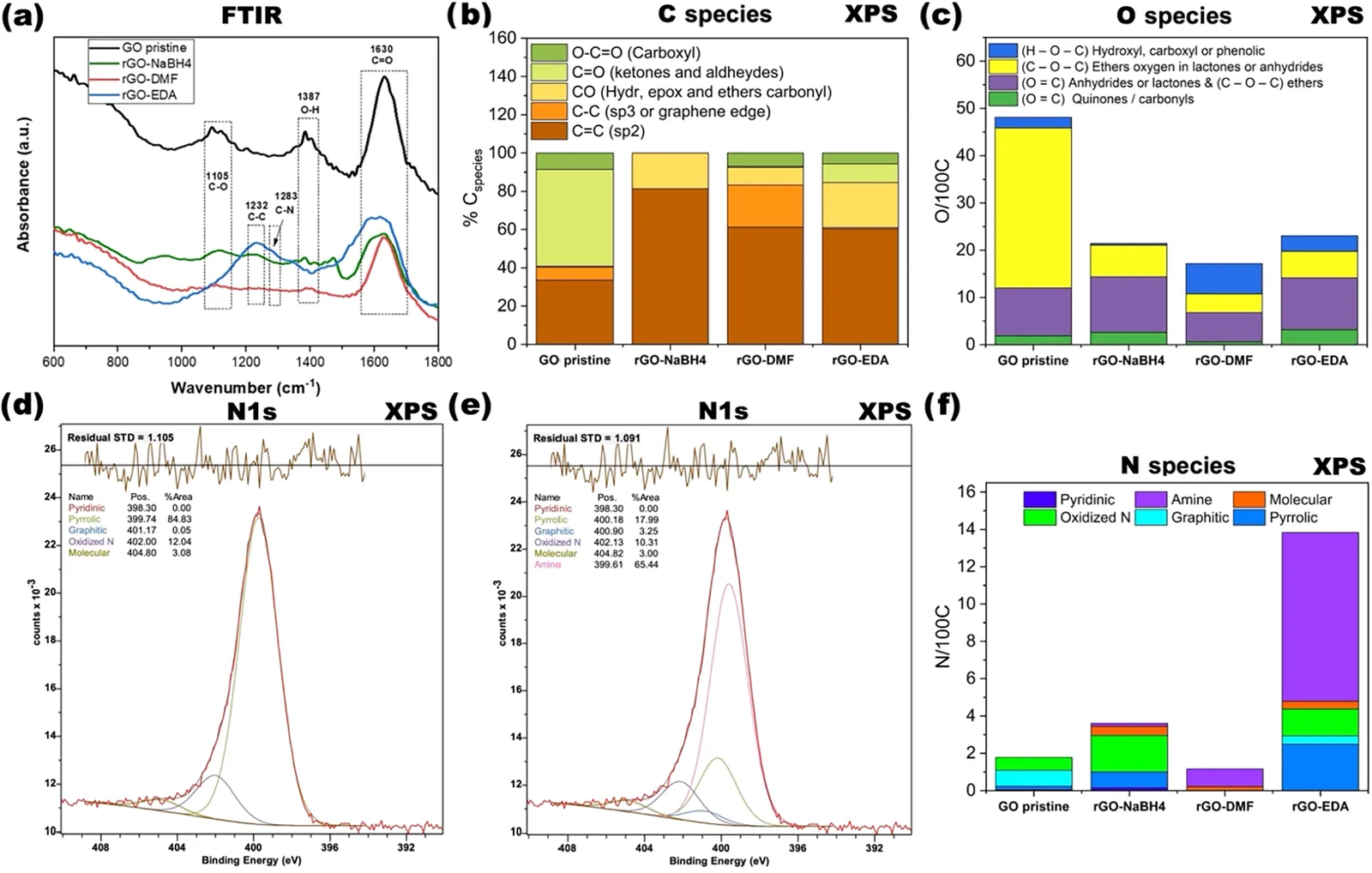
Characterization of the obtained rGO samples. (a) FTIR spectra of pristine GO and the three rGO samples. XPS quantification of samples reduced with NaBH_4_, EDA, and DMF: (b) carbon functional group distribution and (c) oxygen functional group distribution. Panels (d, e) show the deconvolution of the N 1s XPS spectrum of rGO-EDA, considering the commonly reported nitrogen species for N-rGO materials and the same fitting including an additional amine-related component, respectively. (f) Distribution of nitrogen species in GO and the three reduced samples.

Figure 2a shows the FTIR spectra to identify the chemical functional groups present on the rGO samples, obtained after their reduction processes using a temperature of 80 °C as part of the reduction treatments. This figure reveals three main bands at 1105 cm^–1^, 1387 cm^–1^, and 1630 cm^–1^ for the pristine GO, corresponding to C–O, O–H, and C=O bonds, respectively.^63^ These three bands appear in all four samples and are characteristic of oxygen components in GO and remaining oxygen groups in the rGO structures. When comparing these bands across the different samples, a significant decrease in intensity is observed in the rGO-NaBH_4_, rGO-DMF, and rGO-EDA samples compared to pristine GO. This reduction in intensity is attributed to the lower amount of oxygen bonded on the surface due to the chemical reduction with the different reducing agents.

Figure 2a also shows two additional bands at 1232 cm^–1^ and 1283 cm^–1^, attributed to C–C and C–N bond vibrations, respectively.^63^ The C–C bond band is not as evident in the spectrum of pristine GO but becomes more pronounced in the spectra of rGO-NaBH_4_, rGO-DMF, and rGO-EDA, likely due to the high reduction of the pristine GO surface, where C–O vibrations are predominant. The band associated with C–N appears only in the rGO-EDA sample, and it could be associated with the vibration of the bond between the amino group of EDA and graphene sheets (consistent with the ansatz proposed in Figure 1f). This band does not appear in rGO-DMF, probably because DMF molecules exhibit a stronger tendency to interact electrostatically with the GO surface, making the formation of covalent bonds more challenging compared to EDA. Table S1 summarizes the bands present in the different GO and rGO samples.

The samples were characterized by XPS to obtain further information on their chemical composition. Figure 2b shows a summary of the carbon species detected from the high-resolution XPS window of the carbon 1s core level, Figure 2c shows the oxygen species detected from the high-resolution XPS window of the oxygen 1s core level, and Figure 2f shows the nitrogen species detected from the high-resolution XPS window of the nitrogen 1s core level (the graphs with the deconvoluted spectra are shown in Figures S1–S5 in the Supporting Information).

Figure 2b shows the percentage of carbon atoms forming C=C bonds with sp^2^ hybridization, typical of the graphene structure. It can be seen that the pristine GO sample presents around ∼30% of its carbon content with this type of chemical environment, while the reduced samples show ∼80% (rGO-NaBH_4_), ∼60% (rGO-DMF), and ∼60% (rGO-EDA), displaying different degrees of chemical reduction. Interestingly, although rGO-NaBH_4_ has the highest percentage of C=C sp^2^ carbon bonding environment, it contains a similar amount of oxygen groups when compared to rGO-DMF, which possesses the highest percentage of C–C with sp^3^ hybridization bonding (∼20%). Figure 2c, shows, with more precision, the global amount of oxygen atoms detected with respect to carbon as the reference, and their distribution among different oxygen species. It can be noted that in the three reduced samples (rGO-NaBH_4_, rGO-DMF, and rGO-EDA), there is a decrease in the global amount of oxygen groups when compared to the pristine GO sample. Moreover, rGO-DMF shows the smallest amount of oxygen remaining groups, followed by rGO-NaBH_4_ and rGO-EDA. Here, it is important to remember that the reduction process with DMF occurs in the absence of water, avoiding the formation of hydrates on the rGO structure, which must contribute to the result in the lowest amount of oxygen groups. For the rGO-NaBH_4_ and rGO-EDA samples, the NaBH_4_ reducing power is greater than that of EDA.

Subsequently, high-resolution XPS spectra corresponding to the N 1s binding energy region were analyzed considering the commonly reported nitrogen species for N-rGO materials. These common nitrogen species correspond to pyridinic N, pyrrolic N, graphitic N, oxidized N, and molecular N, as shown in the deconvolution performed for the rGO-EDA sample in Figure 2d. However, a very high amount of pyrrolic N (∼85% of the total nitrogen measured) was obtained in the analysis, a very intriguing value not consistent with the experimental conditions performed, since 80 °C during the reduction process does not seem to provide enough energy to incorporate nitrogen atoms and to reconfigure the carbon network of the graphene sheets to obtain such a high amount of pyrrolic nitrogen. Therefore, further analysis was performed to obtain deeper insight into the underlying mechanisms. Exploring the literature and several databases for XPS binding energies of nitrogen groups in organic materials, it was observed that the amine groups signal (398.7 eV–399.9 eV)^37,40,64–66^ could overlap in the binding energy region of pyrrolic nitrogen (399.5 eV–400.3 eV),^37,38,40–42^ a very plausible scenario in our system when using EDA molecules with primary amines at each extreme or with secondary or tertiary amines if the EDA molecules would be grafted to the graphene sheets (as in the case of our initial ansatz in Figure 1f). According to the experimental values reported, the tertiary amine signal is located between 399.7 eV and 399.9 eV,^37,40,65^ secondary amines signal is located between 398.9 eV and 399.9 eV,^37,64^ and primary amines signal is located between 398.7 eV and 399.5 eV;^37,64,66^ and there is binding energy overlapping between the three different amine groups. Therefore, these contributions were grouped into a single component, which was freely fitted between 398.7 eV and 399.9 eV^37,40,64–66^ during the deconvolution procedure and assigned to the amine-related signal. When the amine group component was considered in the XPS analysis, the quality of the fitting was improved, and the pyrrolic N quantity decreased considerably, while the quantity of the amine groups was the predominant nitrogen species assigned (Figure 2e). The results obtained when improving the precision of the XPS analysis of the N 1s peak by taking into consideration the possibility of the presence of amine groups are consistent with the rest of the characterization techniques explored so far and the experimental conditions under consideration, providing further experimental evidence of the grafting of EDA molecules onto graphene sheets during the reduction process. Figure 2f presents a comparison following similar analyses for the nitrogen species present in the rGO-DMF and rGO-NaBH_4_ samples obtained. It can be seen that even when the same conditions were followed for the deconvolution analyses, the presence of amine groups in the rGO-DMF and rGO-NaBH_4_ was very poor when compared to the rGO-EDA sample. Even the pristine GO sample initially contains a small amount of nitrogen (∼2 nitrogen atoms per 100 carbon atoms), presumably inherent to the GO synthesis process.

To provide a clearer point of comparison among the three different samples and to complement our analyses, we also present the XPS survey spectra of the three samples (rGO-NaBH_4_, rGO-DMF, and rGO-EDA) along with the atomic percentage quantification for carbon, nitrogen, and oxygen (see Figure S6). Notably, the rGO-EDA sample exhibited the highest amount of nitrogen compared with the other two samples. We also include magnified views of the lower binding energy regions to account for the purity of the obtained samples (Figure S6d–f).

Moreover, by taking advantage of the atomic percentage quantification from the XPS survey spectra analyses, we are able to estimate the absolute atomic percentage for the different nitrogen species measured in each sample for direct comparison (Table 1). The high content of amines in the rGO-EDA sample after the reduction step clearly stands out.

**Table 1:** Absolute Atomic Percentages of Different Nitrogen Species Measured by High-Resolution XPS Spectra for the Three Different Samples after the Reduction Step

rGO-NaBH_4_		rGO-DMF		rGO-EDA	
nitrogen species	absolute %	nitrogen species	absolute %	nitrogen species	absolute %
Pyridinic N	0.19	Pyridinic N	0.00	Pyridinic N	0.00
Pyrrolic N	1.11	Pyrrolic N	0.00	Pyrrolic N	2.06
Graphitic N	0.00	Graphitic N	0.00	Graphitic N	0.37
Oxidized N	2.53	Oxidized N	0.00	Oxidized N	1.18
Molecular N	0.65	Molecular N	0.11	Molecular N	0.34
Amine	0.22	Amine	0.53	**Amine**	**7.48**

The high nitrogen content detected in the rGO-EDA sample can be attributed to the direct interaction between the EDA molecule and the GO surface during the reduction process, which could be forming a covalent bond at the epoxy group sites on the surface (reinforcing the initial ansatz proposed in Figure 1f), giving rise to the amine group signal detected. In contrast, the rGO-DMF sample presents a considerably lower amount of nitrogen, suggesting that nitrogen from the DMF molecules is not strongly bonded to the graphene sheets during the reduction process, and the weaker interaction between these components may be driven only by charge differences. Figure S7 presents the Raman spectra of rGO-NaBH_4_ and rGO-EDATT800. Both materials exhibit characteristic D and G bands located at approximately 1350 and 1590 cm^–1^, respectively. The D band is associated with defect-induced vibrations arising from structural disorder within the graphitic lattice, whereas the G band corresponds to the in-plane stretching mode of sp^2^-hybridized carbon atoms.^67,68^ The intensity ratio of these bands (I_D/I_G) was used to evaluate the degree of disorder and defect density in the graphene framework. The rGO-EDATT800 sample exhibited a higher I_D/I_G ratio (1.12) than rGO-NaBH_4_ (0.99), indicating an increase in the defect density after thermal treatment at 800 °C. This behavior can be attributed to the partial disruption of the extended sp^2^ domains into smaller graphitic regions and is further consistent with the incorporation of nitrogen atoms into the carbon lattice, which introduces additional lattice distortions. Representative TEM micrographs of rGO-EDATT800 are also shown in Figure S7b, revealing the characteristic sheet-like morphology of graphene. The nanosheets display a wrinkled and corrugated structure, which is commonly observed in thermally treated graphene-based materials, and may be associated with structural rearrangements induced during nitrogen incorporation and high-temperature processing.

To gain deeper insight into the interactions between the EDA or DMF molecules and the GOs, five different GO models were examined (see Figure 3), three of which were previously reported by Paez-Ornelas et al.,^69^ plus the epoxy (C3) and the hydroxyl planar (C5) groups. Figure 3a shows the presence of a hydroxyl group in a single carbon vacancy (C1 model); the C2 model (Figure 3b) represents an oxygen atom as a carbonyl group passivating the C-vacancy; Figure 3c displays an epoxy group (C3 model); a carboxyl group in a single C-vacancy is considered in the C4 model (Figure 3d); and the C5 model corresponds to a planar hydroxyl group (Figure 3e). Figure 3f exhibits the atomistic models of the EDA and DMF molecules.

**Figure 3:**
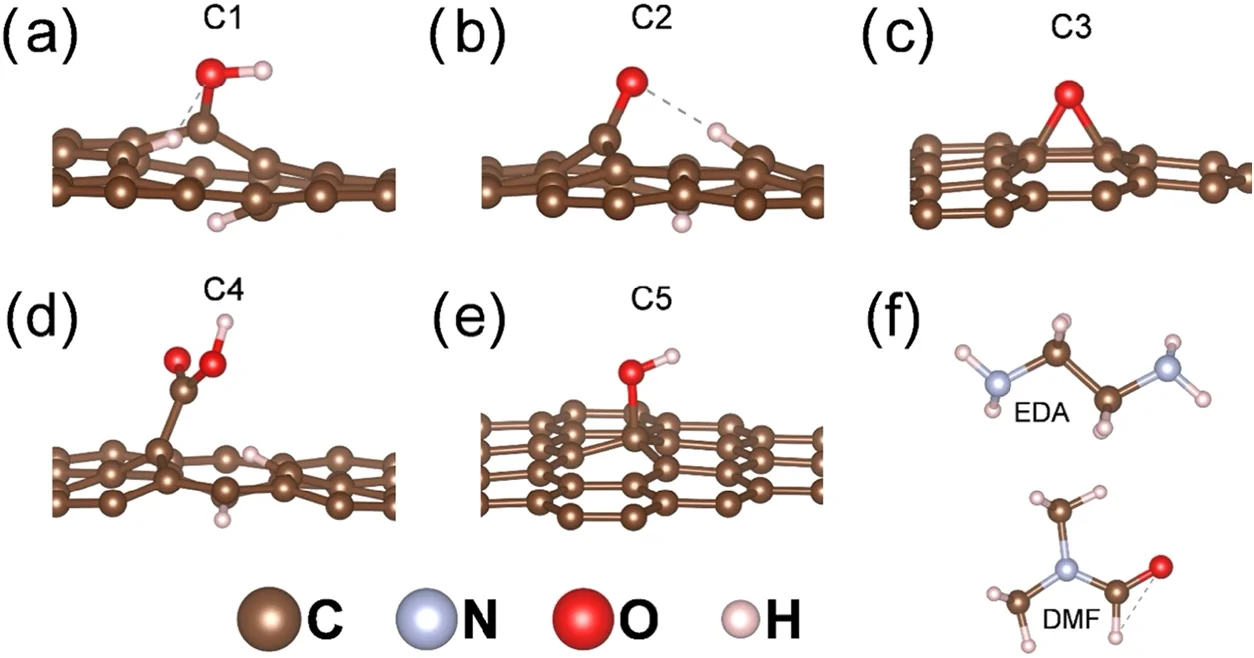
Five different models of GOs considered in this work are labeled as (a) C1, (b) C2, (c) C3, (d) C4, and (e) C5. (f) EDA and DMF molecules are shown.

The adsorption energy of both molecules was calculated by employing the following formula

1 $$E_{\mathrm{ads}}=E^{\mathrm{GO}‐\mathrm{mol}}-E^{\mathrm{GO}}-E^{\mathrm{mol}}$$

where *E* ^GO‑mol^ is the energy of the adsorbed molecules on GO, and *E* ^GO^ and *E* ^mol^ are the energies of the GO and isolated molecules, respectively. According to this definition, negative values suggest favorable adsorption. Additionally, solvation effects can modify interactions between the molecule and substrate, as demonstrated in previous reports.^70^ Nonetheless, as a first approximation, we decided to study EDA and DMF adsorption in vacuum conditions. Table 2 summarizes the adsorption energies (in eV). Several configurations were investigated; however, we have only shown the most stable configuration for each case. Notice that similar trends are observed for both molecules. The most favorable adsorption occurs in the C1 model, corresponding to a hydroxyl functional group, with *E* ^ads^ of −1.02 and −0.89 eV for EDA and DMF, respectively. Then, for EDA, the C4 configuration has an adsorption energy of −0.83 eV, followed by the C5, C3, and C2 models with *E*_ads_ of −0.37, −0.32, and −0.27 eV, respectively. For the DMF case, the C5 model has the second largest energy with −0.67 eV, followed by C4 (−0.56 eV), C2 (−0.38 eV), and C3 (−0.33 eV).

**Table 2:** Adsorption Energies (*E* ^ads^) for EDA or DMF on the Five Different GO Structures Evaluated

	*E*_ads_ (eV)				
molecule	C1	C2	C3	C4	C5
EDA	–1.02	–0.27	–0.32	–0.83	–0.37
DMF	–0.89	–0.38	–0.33	–0.56	–0.67

Figure 4 shows the atomistic representation of the adsorption models of EDA (upper panel) and DMF (lower panel) on GO. The reduced density gradients (***s***) are included to visualize the noncovalent interactions (NCI) that involve such adsorptions. The ***s*** isosurfaces (with ***s** =* 0.5 au) are colored in the RGB scheme (blue, green, and red colors correspond to attractive, van der Waals (vdW), and repulsive interactions, respectively).

**Figure 4:**
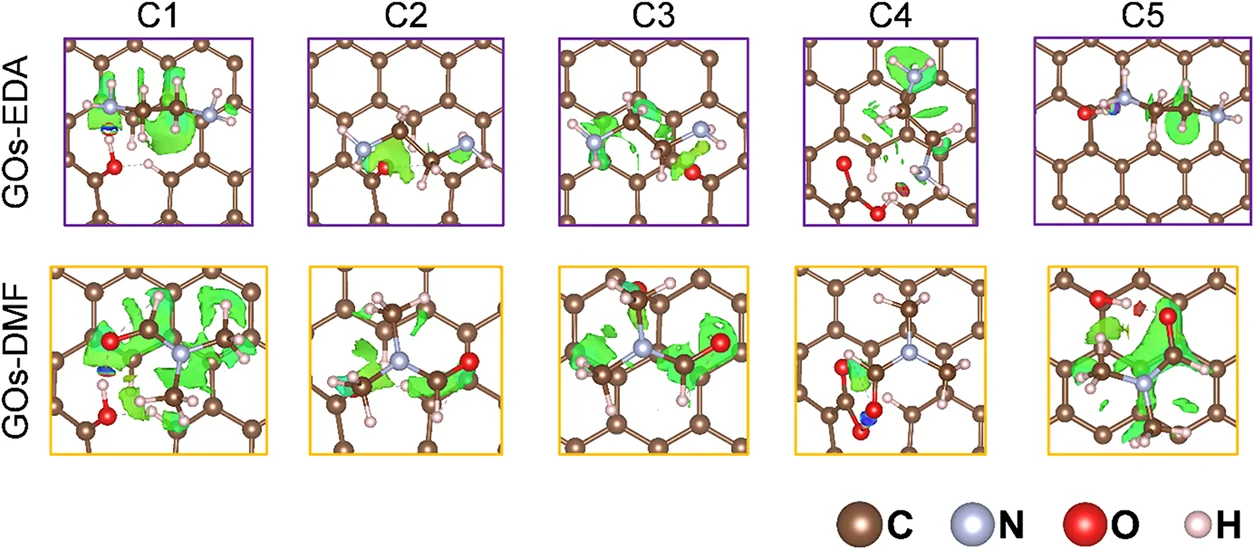
Atomistic representation of the adsorption interactions of EDA or DMF on the five different GO under evaluation. The noncovalent interaction (NCI) index is included by isosurfaces with the RGB color code (with ***s**=* 0.5 a.u.), where blue, green, and red colors correspond to attractive, van der Waals (vdW), and repulsive interactions, respectively.

For EDA adsorption onto GO (upper panel in Figure 4), the C1 and C4 models exhibit a notorious interaction between the amino groups and the hydroxyl and carboxyl groups, respectively. In both cases, the s vs sig­(λ_2_)­ρ graphs (see Figure S8) show sharp peaks located at ∼ −0.1 and ∼0.1 au, which evidence the formation of a bond between the N from the amino group and the H from the O-based functional group. Note that for the C1 model, the sharp peaks are located at larger ρ values in comparison with C4, implying a stronger interaction. This interaction is the reason for the largest adsorption energy values for these two functional groups. Moreover, the C1 model displays how a large portion of the EDA molecule interacts with graphene by vdW forces (green isosurface); this type of interaction is decreased in the C4 case. In the C5 model, hydroxyl and amino groups generate a hydrogen bond (blue isosurface), which exhibits a peak at ±0.05 au (see Figure S8), suggesting a weak interaction between functional groups in comparison with the C1 and C4 models. In contrast, the C2 model exhibits the least interaction between molecules and graphene, consistent with the smallest value of adsorption energy. Regarding C3, the oxygen atom interacts mainly by vdW forces with the EDA molecule, specifically with its nitrogen atom. This is important to note because even when the covalent bond is not obtained as a spontaneous reaction, the positioning and the vdW interaction could aid in covalent bond formation by the injection of enough energy, as the 80 °C employed during the reduction process.

The lower panel in Figure 4 displays the structural models for the adsorption of the DMF molecule onto GO, following the same reduced density gradient isosurfaces (***s**=* 0.5 au) as in the EDA case. As shown in Table 2, the C1 configuration had the largest value of adsorption energy (the most favorable), followed by the C5 and C4 configurations. This can be explained by the hydrogen bond formed (marked by the blue region) between the hydroxyl group (in the carboxyl group) from GO (C1) and the oxygen present in the aldehyde group of the DMF molecule. This assumption is corroborated in the s vs sig­(λ_2_)­ρ graphs (see Figure S8), where the sharp peak located at −0.07 au is associated with this interaction. Furthermore, it is worth noting that in the C1 configuration, the complete DMF molecule interacts with the graphene substrate by vdW forces (marked by the green regions). In the C5, a red zone is observed between the OH and aldehyde group, indicating repulsion. The s vs sig­(λ_2_)­ρ graphs (see Figure S8) show a peak at 0.05 au, corroborating the repulsion between them; however, the complete molecule interacts with GOs by vdW forces, increasing their adsorption energy. On the other hand, in the C4 model, although a hydrogen bond is formed between the H from the O-based group and the aldehyde group from DMF, the molecule does not interact with GO, which is why C5 has a larger adsorption energy. Regarding the C2 and C3 models, the interaction between the oxygen-based functional group from GO and the DMF molecule is carried out mainly through the methyl groups only by vdW interactions, resulting in the lowest adsorption energies. Notably, the predominant interaction of the DMF molecule with the GO models is through the aldehyde group and not by nitrogen participation.

This detailed analysis shows a good interaction between the EDA molecule and some of the oxygen groups present in pristine GO, particularly hydroxyl-containing groups, which promote intense interactions with amino groups. However, in all cases, the O-based functional groups work as active sites to trap the molecules and could promote the formation of nitrogen-doped graphene. These results suggest possible covalent-anchoring events between EDA molecules and rGO induced by the thermal energy supplied during the reduction process at 80 °C, consistent with the spectroscopic signals associated with nitrogen incorporation in the rGO-EDA sample (Figure 2a,2e,2f).

Furthermore, the rGO-EDA sample showed unexpected behavior when monitored by UV–visible spectroscopy in its reduction process (see Figure 1c,1e), with a blue shift of the absorbance peak, while rGO-NaBH_4_ and rGO-DMF showed the conventional red-shifting of their absorbance peaks. However, the chemical characterization showed a reduction due to the decrement in oxygen group signals (Figure 2). Then, our initial ansatz (see Figure 1f) was explored by first-principles calculations evaluating the effect of the adsorbed molecules on the electronic properties of rGO by calculating the band structure along the Γ–M–K−Γ path from the Brillouin zone. In all cases, the energy reference is set to the Fermi level. Figure S9 (Supporting Information) shows the band structure of the five different models of GO in Figure 3 without adsorbed molecules. Results show that oxygen functional groups modify the band gap. Models C1, C2, C4, and C5 have directed band gaps of 0.46, 0.55, 0.47, and 0.39 eV, respectively, located at the *K* point. On the other hand, in the epoxy group (C3 model), the Dirac cone remains with a slight displacement from the *K* point for pristine graphene to the K−Γ trajectory.

Figures S10 and S11 depict the band structure when EDA or DMF molecules are adsorbed onto different GO models. For both molecules, flat bands below the Fermi level, related to adsorbed molecules, were observed. However, the shapes of the maximum valence band (MVB), minimum conduction band (MCB), and energy gap are not altered significantly. In the experiment, it is important to remember that during the reduction processes, a red shift in the UV–visible spectra for the rGO-NaBH_4_ sample (Figure 1) is observed, which can be indirectly related to a decrement in the energy gap as the reaction time increases, evidencing the chemical reduction of the pristine GO sample. However, this behavior is not consistently observed for the different models under evaluation when EDA and DMF are adsorbed. Therefore, adsorption is not the only interaction present between GO and the EDA or DMF molecules during the experiments. Then, the effect of functional group concentration on the electronic properties of GO was considered using epoxy (C3) and planar hydroxyl (C5) groups as model systems.

Figure 5a shows the band diagram plots for the epoxy (C3) and OH planar (C5) groups in GO. The presence of one (left panel) or two (right panel) functional groups per supercell was considered. The structural models are shown in the lower panel of Figure 5a. It can be observed that one epoxy group per supercell is not enough to open the gap, and the Dirac cone remains preserved. However, when two epoxy groups are considered, a band gap of 0.18 eV appears. Regarding the hydroxyl groups, one functional group per supercell exhibits a band gap of 0.39 eV, and when two functional groups are considered, the band gap increases to 0.58 eV. These calculations are consistent with how the decrement of oxygen-based functional groups decreases the band gap of GO when it is chemically reduced into rGO, in agreement with previous reports.^71,72^ Moreover, this is consistent with the conventional red shift of the absorbance peak observed in the UV–visible spectra when using a common reductant molecule such as NaBH_4_.

**Figure 5:**
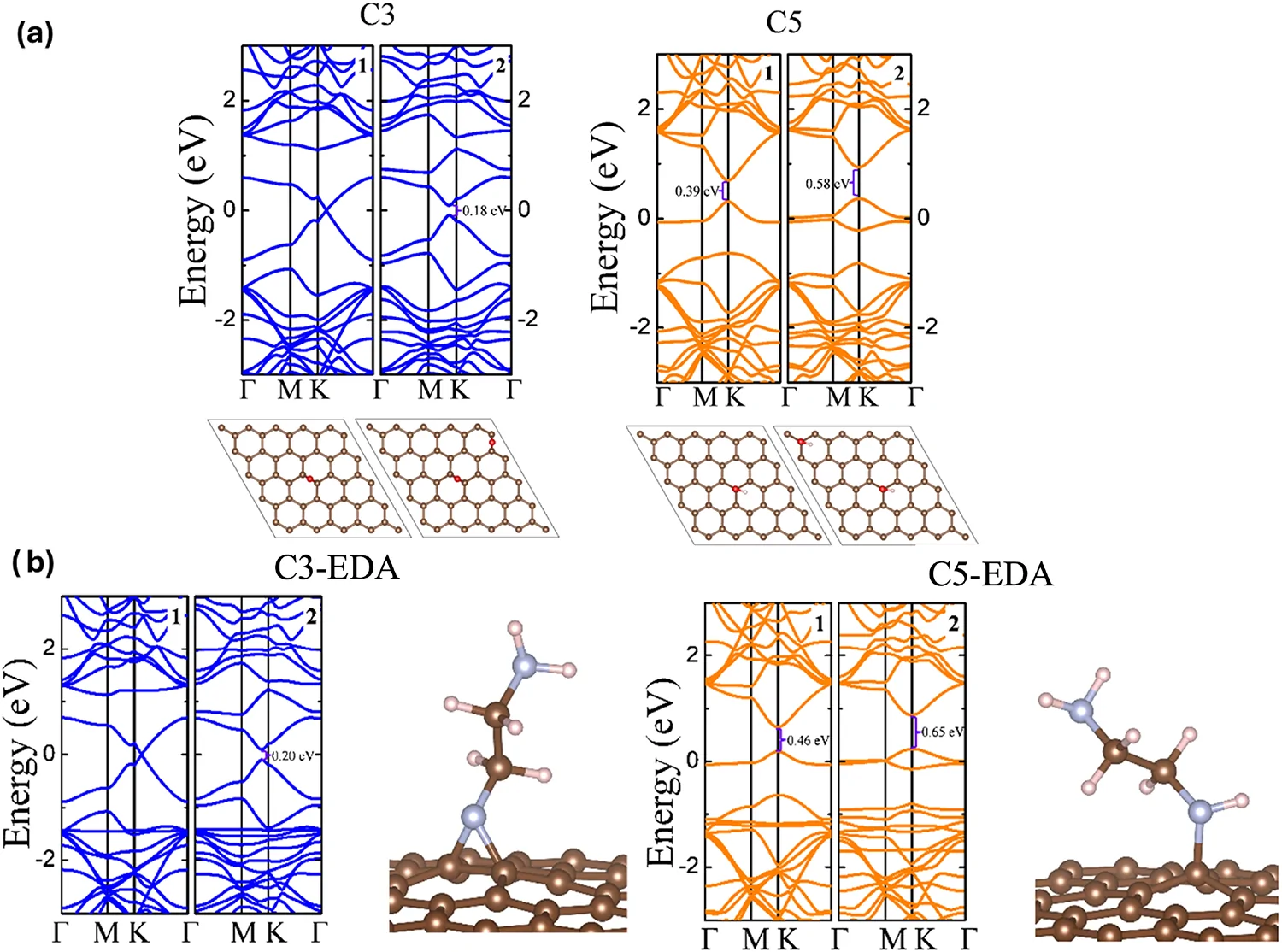
(a) Band structures of the C3 (epoxy) and C5 (hydroxyl planar) models with different functional group concentrations; atomic models are included in the lower panel. (b) Band structures along the Γ–M–K−Γ pathway for the chemisorption of EDA molecules onto the graphene sheets by replacing one or two epoxy groups (C3 model) in the left panel or one or two hydroxyl groups (C5 model) in the right panel; the atomistic representation of the anchoring molecule models is included on their right side.

However, the last results do not yet explain the blue-shifting observed for the case of the rGO-EDA sample. Further calculations were performed with the chemisorption of the EDA molecule on the GO, considering the same C3 and C5 models. For the epoxy group (C3), EDA is attached to GO by removing the two hydrogen atoms of one of the amino groups of the EDA molecule and the oxygen-based functional groups of the GO, as in the initial ansatz proposed in Figure 1f. This causes the formation of two N–C bonds of the EDA with the graphene sheet with a bond distance of 1.41 Å (see Figure S12 with the relaxed model), generating a tertiary amine group. The band structure of this C3-EDA model is displayed in Figure 5b for one (left) and two (right) functional groups per supercell. When only one EDA molecule is chemisorbed onto GO, the Dirac cone is preserved, similar to the C3 model (Figure 5a). Once two molecules are attached to the GO, a direct band gap of 0.20 eV near the K point appears, which is 20 meV larger than the band gap for two epoxy groups per supercell. This is an important result, evidencing that the interchange of epoxy groups by the chemisorption of EDA molecules during the reduction process could change the electronic structure of the rGO-EDA, accounting for the blue-shifting observed in the UV–visible spectra.

Moreover, for the hydroxyl group (C5), chemisorption can occur by removing one hydrogen from the amino group of the EDA molecule and one OH from the GO. This results in the covalent attachment of EDA to the graphene sheet by one C–N bond with 1.52 Å (see the relaxed model C5-EDA in Figure S12), generating a secondary amine group. The C5-EDA model’s band structures for one (left) and two (right) chemisorbed EDA are shown in Figure 5b. Direct band gaps of 0.46 and 0.65 eV are obtained for each case. Once more, the band gap (0.65 eV) is 70 meV larger than the band gap (0.58 eV) of the progenitor structure (see model C5 in Figure 5a). This reinforces the plausibility that the blue-shifting observed in the UV–visible spectra appears from the interchange of the oxygen-based groups (epoxy oxygen (C3) or hydroxyl group (C5)) by the chemisorption of the EDA molecules during the reduction process.

As a proof of concept, we performed NEB calculations to estimate the activation energies required to form covalent bonds between EDA and GO. The results are shown in Figure S13 in the SI. The initial stage (IS) is the energy reference and corresponds to the adsorption of EDA on the epoxy group. The final stage (FS) occurs when the amide group donates a proton to the epoxy group, forming a hydroxyl group and a covalent bond with GO. The activation energy of the process is 0.44 eV, corresponding to the transition state (TS), at which point proton exchange between EDA and the epoxy group occurs.

Once the blue-shifting observed in the UV–visible spectra for the rGO-EDA during the reduction process and the nitrogen presence observed in this sample with the FTIR and XPS analyses were understood, a high content of EDA molecules anchored to the graphene sheets was assumed for the functionalized rGO-EDA. Therefore, heat treatment at 800 °C under an inert atmosphere was applied to promote the reconfiguration of the carbon network within the graphene sheets, facilitating the incorporation of nitrogen atoms into the carbon framework.

Figure 6 shows the FTIR spectra (Figure 6a), and the distribution of carbon groups (Figure 6b) and oxygen groups (Figure 6c) obtained from the XPS signal, together with the deconvolution of the N 1s XPS spectra corresponding to the rGO-EDA_TT800 °C sample considering the common nitrogen species present in N-rGO samples (Figure 6d) or including an extra component to account for the presence of amine groups (Figure 6e). The distribution of nitrogen species in the different samples under evaluation after the 800 °C heat treatment is presented in Figure 6f.

**Figure 6:**
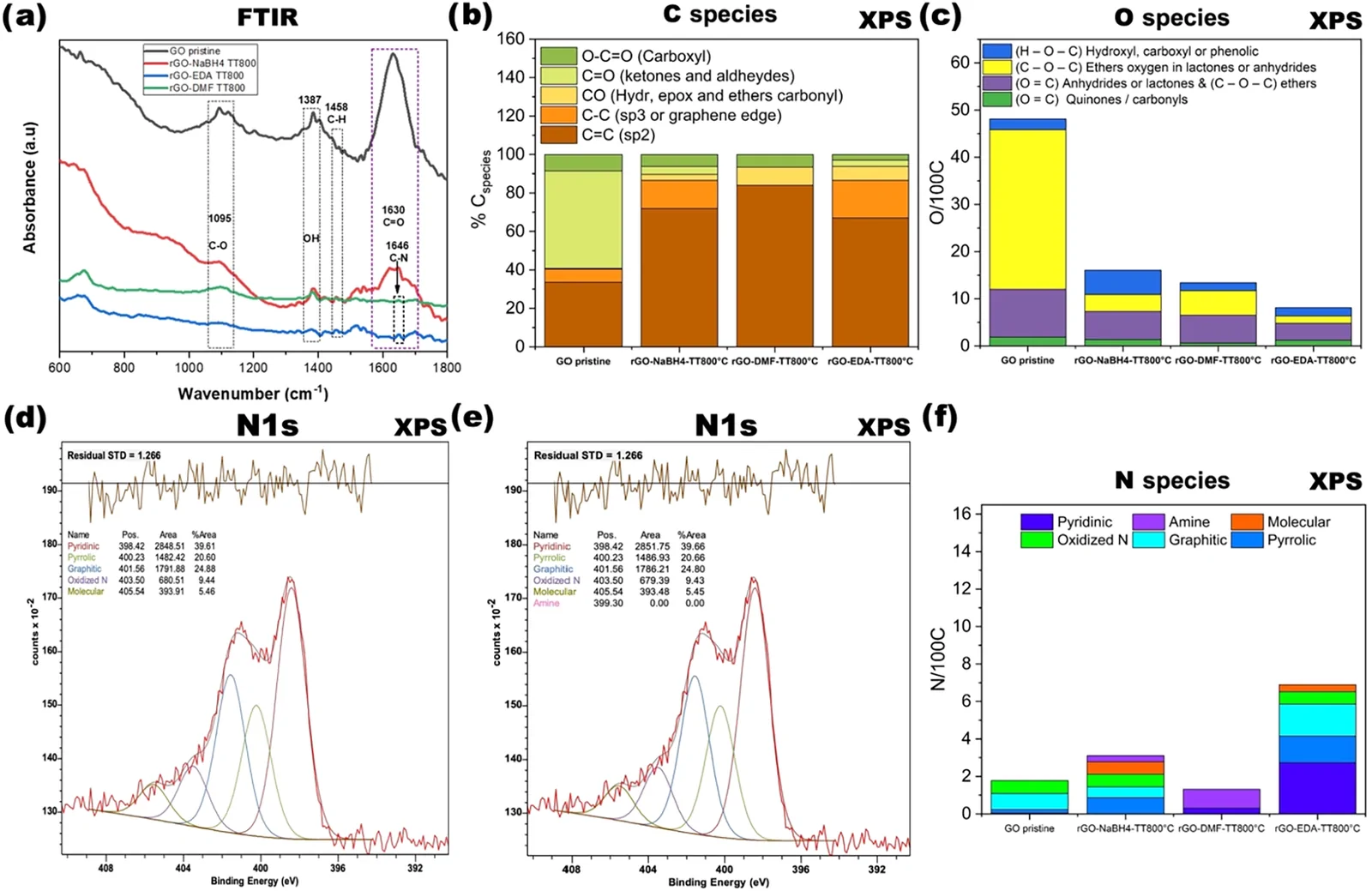
Characterization of the rGO samples obtained after heat treatment at 800 °C: (a) FTIR spectra of the pristine GO sample as a reference and the three rGO samples obtained after heat treatment at 800 °C. XPS quantification of the different obtained samples: (b) carbon group distribution, (c) oxygen group distribution, (d, e) deconvolution of the N 1s XPS data from sample rGO-EDA_TT800 °C by the common nitrogen species considered for N-rGO and including an additional component to consider the presence of amine groups, respectively; and (f) nitrogen groups distribution for the GO sample and the three obtained samples.

Figure 6a shows the FTIR spectra of the rGO-NaBH_4_, rGO-DMF, and rGO-EDA samples obtained after heat treatment at 800 °C for 3 h, together with the pristine GO sample as a reference. Two main bands are observed in all four spectra at 1095 cm^–1^ and 1387 cm^–1^, corresponding to C–O and O–H bond vibrations, respectively. Remnants of oxygen persist on the rGO surfaces after thermal treatment. At 1458 cm^–1^, the band from the C–H vibration can be barely perceived. Similarly, a small signal can be perceived at 1646 cm^–1^ in the rGO-EDA and rGO-DMF samples, associated with the C–N bond vibrations.^73^ This signal appears in the region of the C=O band (1630 cm^–1^), where these two samples had a higher C=O band signal before the 800 °C heat treatment (see Figure 2a). Remarkably, the C=O band signal (1630 cm^–1^) almost disappears when compared to the signal from these two samples before the 800 °C heat treatment (see Figure 2a); while the rGO-NaBH_4_ sample maintains some signal from this C=O band, but with a considerable decrement as well. Finally, the absence of signals from the C–C band (1232 cm^–1^) and the C–N band (1283 cm^–1^) after the 800 °C heat treatment should be noted (see Figure 6a), while the rGO-EDA sample before the 800 °C heat treatment presented a considerable signal for these two bands (see Figure 2a). This is presumably attributable to the presence of EDA molecules anchored on the graphene sheet prior to the 800 °C heat treatment and their subsequent degradation/reconfiguration after the treatment. Table S2 summarizes the bands measured after heat treatment at 800 °C for 3 h.

In Figure 2b, it can be observed that after heat treatment at 800 °C, the rGO-EDA sample increases the C=C (sp^2^) carbons from ∼60% (see Figure 2b) to ∼68% (Figure 6b) due to the reconfiguration of the carbon network during annealing, while the C–C (sp^3^) carbon increases from ∼0% to ∼18%. In contrast, the rGO-DMF sample exhibits an increase in carbon C=C (from ∼60% to ∼85%), alongside a decrease in carbon C–C (from ∼20% to ∼0%) after 800 °C heat treatment. This could be attributable to the differences in the interaction between the EDA molecules and the DMF molecules with the graphene sheets, since the EDA molecules are attached covalently to the graphene sheets, resulting in a more difficult reconfiguration for their carbon network, while the absence of covalent interaction with the DMF molecules avoids the presence of functionalizing molecules to be incorporated during the annealing.

Figure 6c shows the oxygen species remaining after the 800 °C heat treatment, evidencing a further thermal reduction with annealing when compared with the oxygen present before the 800 °C heat treatment (Figure 2c). The full deconvoluted set of data is shown in Figures S14–S16 (Supporting Information) for the three different samples. Figure 6d,6e shows the comparison of deconvolution of the N 1s XPS data for the rGO-EDA_TT800 °C sample by the five nitrogen species commonly considered for N-rGO samples or by including an additional component to consider the amine group signals, respectively; it can be clearly seen that no signal corresponding to amine groups was detected. This result extends the level of confidence to our analyses because, following the same approach as in Figure 2, no amine groups were detected in the scenario where the sample was exposed to an 800 °C thermal treatment. Figure 6f presents a comparison of the nitrogen species present in the three samples under evaluation, rGO-NaBH_4__TT800 °C, rGO-DMF_TT800 °C, and rGO-EDA_TT800 °C, after the thermal treatment, together with pristine GO as a reference. Even when there was a decrease in N species for the three obtained samples, rGO-EDA_TT800 °C presents a considerably higher amount of nitrogen and no amine groups, reinforcing the idea of the incorporation of nitrogen into the carbon network of the graphene sheets during the 800 °C thermal treatment.

To provide a clearer point of comparison among the three different samples after the 800 °C thermal treatment and to complement our analyses, we also present the XPS survey spectra of the three samples (rGO-NaBH_4_-TT800 °C, rGO-DMF-TT800 °C, and rGO-EDA-TT800 °C) along with the atomic percentage quantification for carbon, nitrogen, and oxygen (see Figure S17). Significantly, a considerable amount of nitrogen is still present in rGO-EDA TT800 °C after thermal treatment. We also include magnified views of the lower binding energy regions to account for the purity of the obtained samples (see Figure S17d–f).

For direct comparison, we estimated the absolute atomic percentage for the different nitrogen species measured in each sample after the 800 °C thermal treatment (see Table 3). Remarkably, the amount of amine nitrogen vanishes while the pyridinic, pyrrolic, and graphitic nitrogen increase considerably. This provides important experimental evidence of the incorporation of nitrogen into the rGO lattice after thermal treatment.

**Table 3:** Absolute Atomic Percentages for the Different Nitrogen Species Measured by High-Resolution XPS Spectra for the Three Different Samples after the 800 °C Thermal Treatment

rGO-NaBH_4_-TT800 °C		rGO-DMF-TT800 °C		rGO-EDA-TT800 °C	
nitrogen species	absolute %	nitrogen species	absolute %	nitrogen species	absolute %
Pyridinic N	0.00	Pyridinic N	0.36	**Pyridinic N**	**2.91**
Pyrrolic N	0.74	Pyrrolic N	0.00	**Pyrrolic N**	**1.52**
Graphitic N	0.49	Graphitic N	0.00	**Graphitic N**	**1.82**
Oxidized N	0.58	Oxidized N	0.00	Oxidized N	0,69
Molecular N	0.56	Molecular N	0.00	Molecular N	0,40
Amine	0.28	Amine	1.19	**Amine**	**0.00**

Using AIMD, we investigate the thermal stability of graphitic- and pyridinic-nitrogen-doped graphene. Our results demonstrate that N-doped graphene samples are stable at 800 °C; see Figure S18 in the SI. After the simulation, nonbroken bonds were observed, indicating their stability, and a slight undulation was also observed. Additionally, we calculated the band structure along the Γ–M–K−Γ path for the models obtained using DFT at 0 K (blue lines) and for the models after AIMD simulations (red lines). Our findings demonstrate that the electronic structure is not modified by temperature. Also, our results agree with those of the previous reports.^74,75^ N-graphitic species shift the Dirac cone to lower energies, whereas N-pyridinic species open and shift the Dirac cone to higher energies.

So far, the 800 °C heat treatment has resulted in a beneficial step toward nitrogen doping of the rGO samples, specifically for the rGO-EDA_TT800 °C sample. Harnessing the high amount of EDA molecules retained on the graphene surface, due to the efficient functionalization during the first chemical reduction process, the subsequent annealing at 800 °C was able to reconfigure the carbon network from the graphene sheet and incorporate a high amount of nitrogen atoms. Moreover, it is known that the nitrogen sites in nitrogen-doped rGO (N-rGO) can induce a local redistribution of charge due to the nitrogen electronegativity, producing an active local region or an active site for electrochemical reactions such as the Oxygen Reduction Reaction (ORR).^8,20,30^ Therefore, to evaluate nitrogen atom incorporation into the graphene network via structural reconfiguration during the 800 °C thermal treatment, the ORR activity of the rGO samples was investigated. This evaluation is intended as a proof of concept to demonstrate that the treatment imparts electrochemical activity to rGO.

Cyclic voltammetry (CV) was initially performed for the rGO-NaBH_4_ (Figure 7a), rGO-EDA (Figure 7b), and rGO-DMF (Figure 7c) samples. Each figure shows three voltammograms: the black curve corresponds to measurements taken while saturating with argon; the red curves represent measurements made while saturating with molecular oxygen for the rGO samples before the 800 °C thermal treatment; and the blue curves represent measurements made while saturating with molecular oxygen for the rGO samples after thermal treatment at 800 °C. It can be observed that the rGO samples before the 800 °C thermal treatment show no electrochemical activity for the ORR (red curves). Even with the significant amount of Nitrogen measured for the rGO-EDA sample (see Figure 2 and Table 1), due to the functionalization of EDA molecules by covalent bonding without incorporation of the Nitrogen into the Carbon network of the graphene sheets. After the 800 °C thermal treatment of the rGO samples, the characteristic signal of oxygen reduction becomes evident predominantly for the rGO-EDA sample with an onset potential at 0.76 V vs RHE (see blue curves). This result reinforces the fact that nitrogen is incorporated into the carbon network as active electrochemical sites. Nonetheless, it should be mentioned that the increment of nonfaradaic current is associated with the three rGO samples.

**Figure 7:**
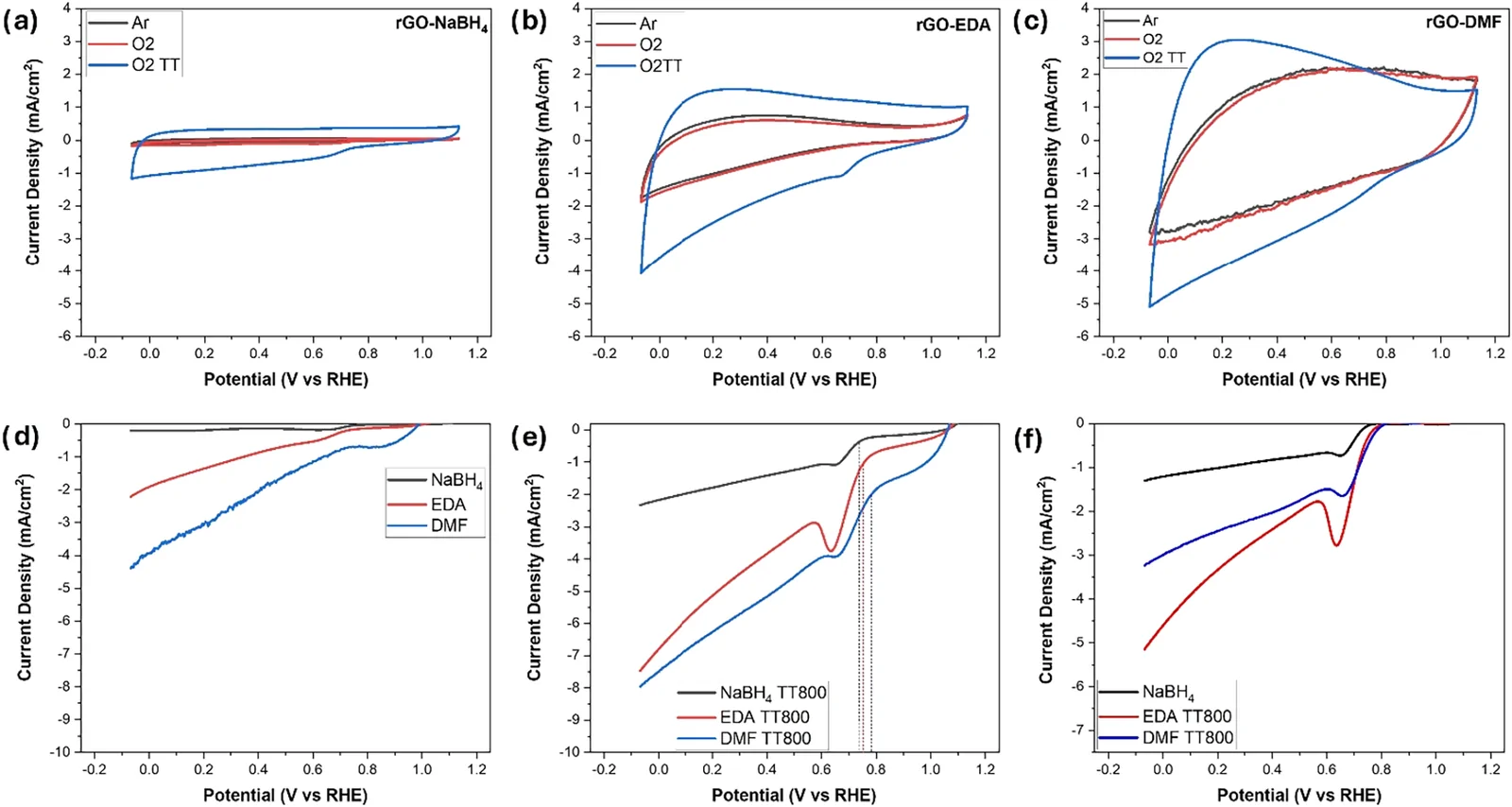
Electrochemical evaluation by cyclic voltammetry of (a) rGO-NaBH4, (b) rGO-EDA, and (c) rGO-DMF samples before and after thermal treatment at 800 °C. (d) Present linear sweep voltammetry measurements (1600 rpm) of the three different samples before and (e) after thermal treatment at 800 °C; (f) same LSV measurements from (e) after the nonfaradaic current subtraction.

Afterward, linear sweep voltammetry (LSV) measurements with a rotating disk electrode (RDE) at 1600 rpm were performed for the rGO samples before (Figure 7d) and after (Figure 7e) the 800 °C thermal treatment. A drastic increase in current density is observed for the rGO-EDA and rGO-DMF samples after the 800 °C heat treatment. Most importantly, a marked peak between 0.6 and 0.7 V vs RHE appears after the 800 °C heat treatment, characteristic of the ORR. This ORR peak appears considerably more prominent for rGO-EDA_TT800 °C. Figure 7e shows a more defined peak for the rGO-EDA sample originating from the baseline of the nonfaradaic current. A small shift of the onset potential toward more positive potentials was also be observed for both samples, rGO-DMF (∼0.78 V vs RHE) and rGO-EDA (∼0.75 V vs RHE), with respect to the rGO-NaBH_4_ (∼0.74 V vs RHE) reference sample. This could be related to the amount of nitrogen incorporated into the rGO-EDA sample (see Table 3), but at the same time correlated with the concentration of structural defects and the electrical conductivity properties of the sample (see Figures 6b and S7). Nonetheless, the peak current registered originating from the nonfaradaic current baseline is higher for the rGO-EDA sample due to a higher reduction event activity of molecular O_2_ at the electrode surface. To make a clearer representation of these results, Figure 7f presents the LSV data from Figure 7e once the nonfaradaic current baseline has been subtracted. Table S3 presents the current density values for the peak current measured from all the three rGO samples in Figure 7e,7f. Even so, the more positive onset potential for the rGO-DMF_TT800 °C sample could be due to the higher amount of sp2 carbon regions along the graphene sheets, according to the XPS data shown in Figure 6b, aiding in the electrical conductivity properties of the sample. To contextualize the obtained onset potential values with previously reported data, a comparison is provided in Table S4 in the Supporting Information.

The electrochemical activity for the ORR, with a drastic improvement after the 800 °C heat treatment, shows how the graphene network reconfiguration was able to incorporate nitrogen sites into the graphene sheets, resulting in nitrogen-doped rGO (N-rGO) samples with electrochemical activity.

## 4 Conclusions

The chemical reduction of GO using reducing agents with nitrogen content was harnessed to obtain N-rGO. Two reducing molecules were tested: DMF and EDA. Notably, the 80 °C reduction process with EDA resulted in the covalent functionalization of the graphene sheets with EDA molecules in the rGO-EDA sample. Moreover, several signatures from the grafting of the EDA functionalizing the rGO-EDA sample were revealed by XPS, FTIR, and UV–visible spectroscopy measurements and by first-principles calculations.

Experimental evidence shows the appearance of a C–N band (∼1283 cm^–1^) in the FTIR spectra, and an amine group signal was detected in the XPS analyses for the rGO-EDA sample. When complemented by the detailed insight from the DFT calculation analyses, the covalent bonding of the EDA molecules to the graphene sheets (see C3 or C5 model transformed into the C3-EDA or C5-EDA models in Figures 3 and 5 presenting tertiary and secondary amine groups) can cause the opening of the energy band gap of the nanostructures, which could account for the blue-shifting observed in the UV–visible spectra peak during the chemical reduction process of the rGO-EDA sample. It is important to note that solvation effects play a significant role in the adsorption energies and activation barriers. In this work, we focus on interactions at vacuum conditions as a first approximation; therefore, it is important to perform future calculations to evaluate solvation effects and their modifications in adsorption and activation energies.

A subsequent 800 °C heat treatment under an inert atmosphere was performed on the obtained rGO samples for the reconfiguration of the carbon network from the graphene sheets and the incorporation of nitrogen as a dopant in the graphene sheets. Moreover, the achieved N-doping confers electrochemical activity to the N-rGO sample, as demonstrated by proof-of-concept ORR testing following the 800 °C heat treatment. Notably, the most prominent peak of the ORR electrochemical activity was obtained for the rGO-EDA_TT800 °C sample.

Hence, the chemical reduction and functionalization of rGO from GO by the EDA reductant molecule, with a subsequent 800 °C heat treatment, results in an efficient approach to obtain N-doped rGO (N-rGO) with electrochemical activity. This opens the possibility to explore a myriad of N-containing molecules by a two-step N-doping process of rGO to harness its characteristic properties in several fields, including energy-related applications.

## Supplementary Material



## Data Availability

The data supporting this article are included as part of the Supporting Information or directly in this main manuscript.

## Notes

FTIR peak assignment values of rGO samples before thermal treatment and of N-rGO samples after the 800 °C thermal treatment; deconvolution of all the XPS spectra data; XPS survey spectra showing the identified byproduct traces of the rGO and N-rGO samples; representative Raman spectra and TEM images of the rGO samples; noncovalent interactions graphs; band structure results for the described models; relaxed structural models for the chemisorbed EDA molecule onto the optimized models; minimum energy pathway for the covalent bond formation between EDA and GO; AIMD results for graphitic and pyridinic N-doped graphene; peak current measured for the rGO samples after the 800 °C thermal treatment; a comparison table with onset potential data for the previously reported ORR (PDF)
