# Supplementary material for: Two-Step Approach to Obtain Nitrogen-Doped Reduced Graphene Oxide Using Nitrogen-Containing Reducing Agents
Source: ACS Phys Chem Au. 2026 Aug 6;6(5):1115–30. doi: 10.1021/acsphyschemau.6c00049 (PMC13614139; doi:10.1021/acsphyschemau.6c00049)
Supplement: Supplementary file 1 [file pg6c00049_si_001.pdf]

Supplementary Information

A two-step approach to obtain Nitrogen-doped reduced Graphene Oxide using Nitrogen-containing reducing agents

Carlos Belman-Rodriguez[a], Rodrigo Ponce-Pérez[a], Jose Ruiz-Marizcal[b,a], Gabriel Alonso-Nunez[a], Jose M. Romo-Herrera[a*]

[a] *Centro de Nanociencias y Nanotecnología, Universidad Nacional Autónoma de México (CNyN-UNAM). Km. 107 Carretera Tijuana-Ensenada, Ensenada, Baja California, México, 22860*

[b] *Posgrado en Nanociencias. CICESE / CNyN-UNAM. Km 107 Carretera Tijuana-Ensenada, Ensenada, Baja California, México, 22860*

e-mail: jmromo@ens.cnyn.unam.mx

Table S1. FTIR peak assignment of rGOs before thermal treatment

Before Thermal Treatment	
Wavenumber (cm^{-1})	Band Assigment
1105	C-O
1232	C-C
1283	C-N
1387	O-H
1630	C=O

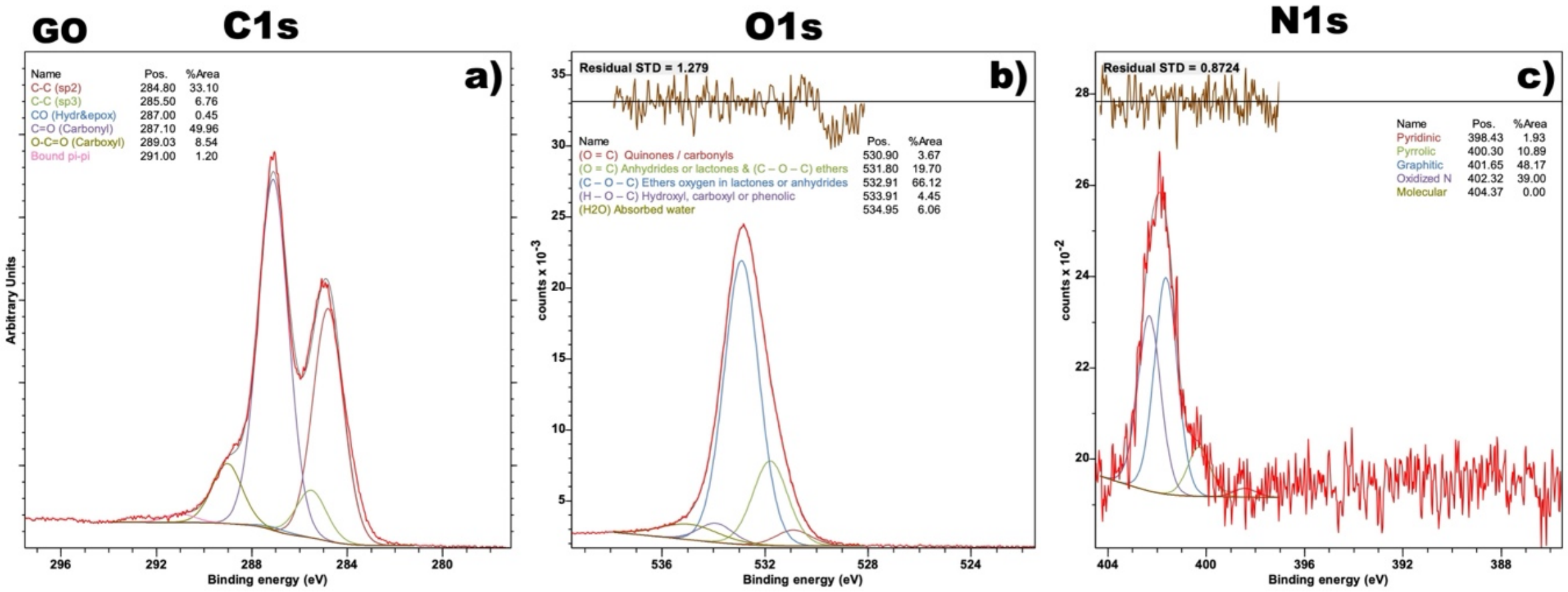

Figure S1. Deconvolution of the XPS spectra corresponding to the a) C1s data, b) O1s data and c) N1s data for the **GO pristine** sample.

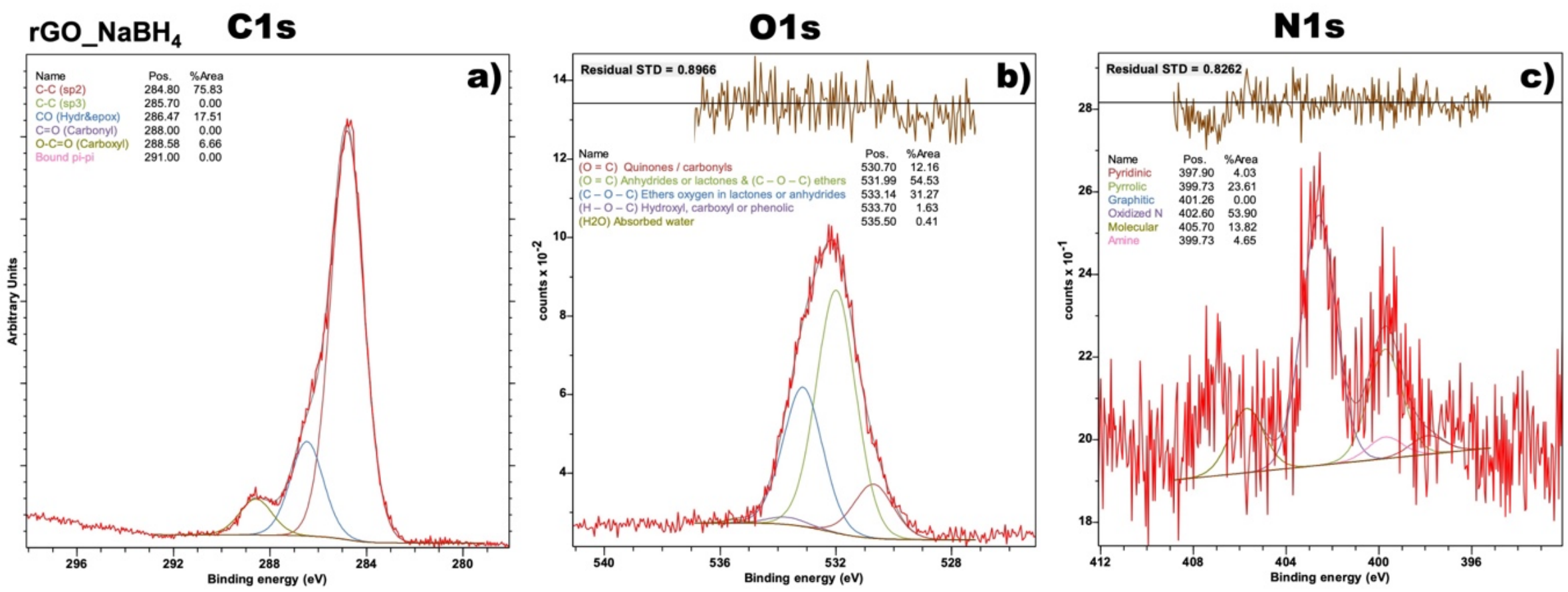

Figure S2. Deconvolution of the XPS spectra corresponding to the a) C1s data, b) O1s data and c) N1s data for the **rGO-NaBH$_4$** sample.

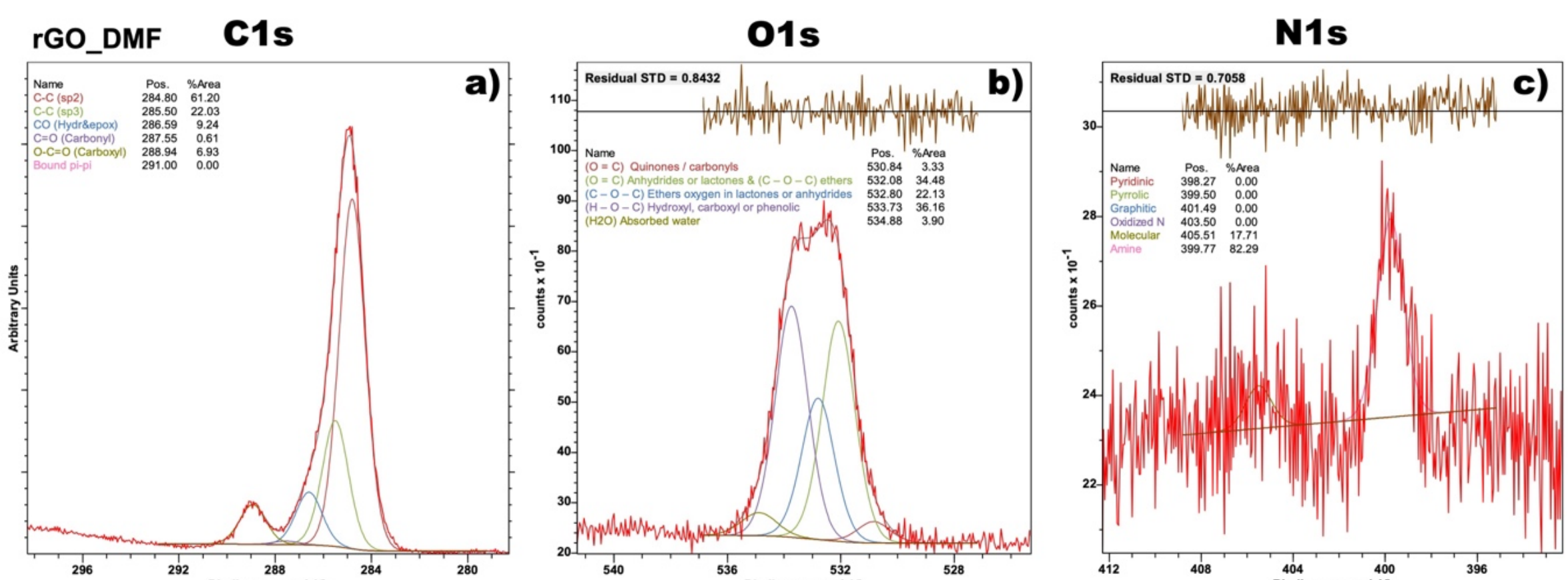

Figure S3. Deconvolution of the XPS spectra corresponding to the a) C1s data, b) O1s data and c) N1s data for the **rGO-DMF** sample.

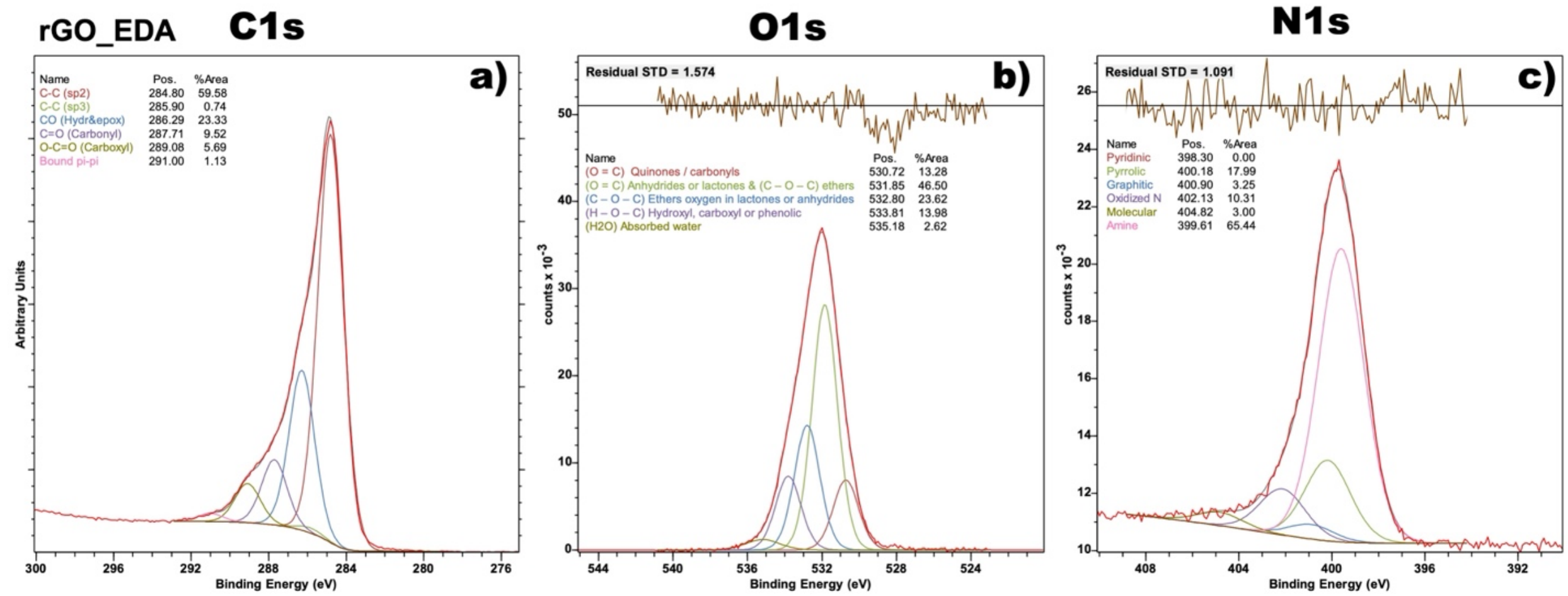

Figure S4. Deconvolution of the XPS spectra corresponding to the a) C1s data, b) O1s data and c) N1s data for the **rGO-EDA** sample.

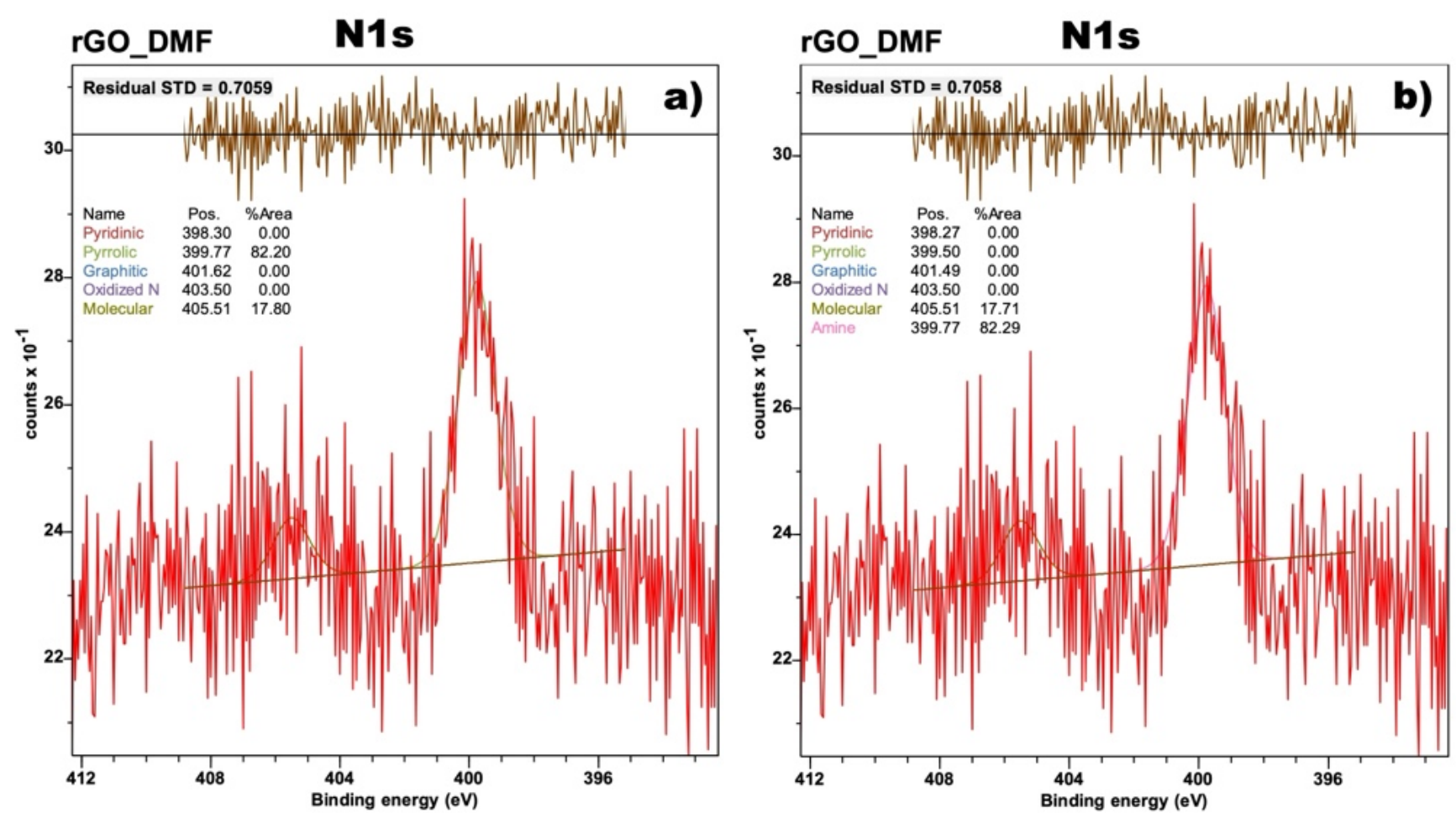

Figure S5. Deconvolution of the XPS spectra for the N1s data for the rGO-DMF sample a) without consideration of the Amine groups component, and b) considering the Amine groups component; increasing the quality of the fitting curve when considering the Amine groups component.

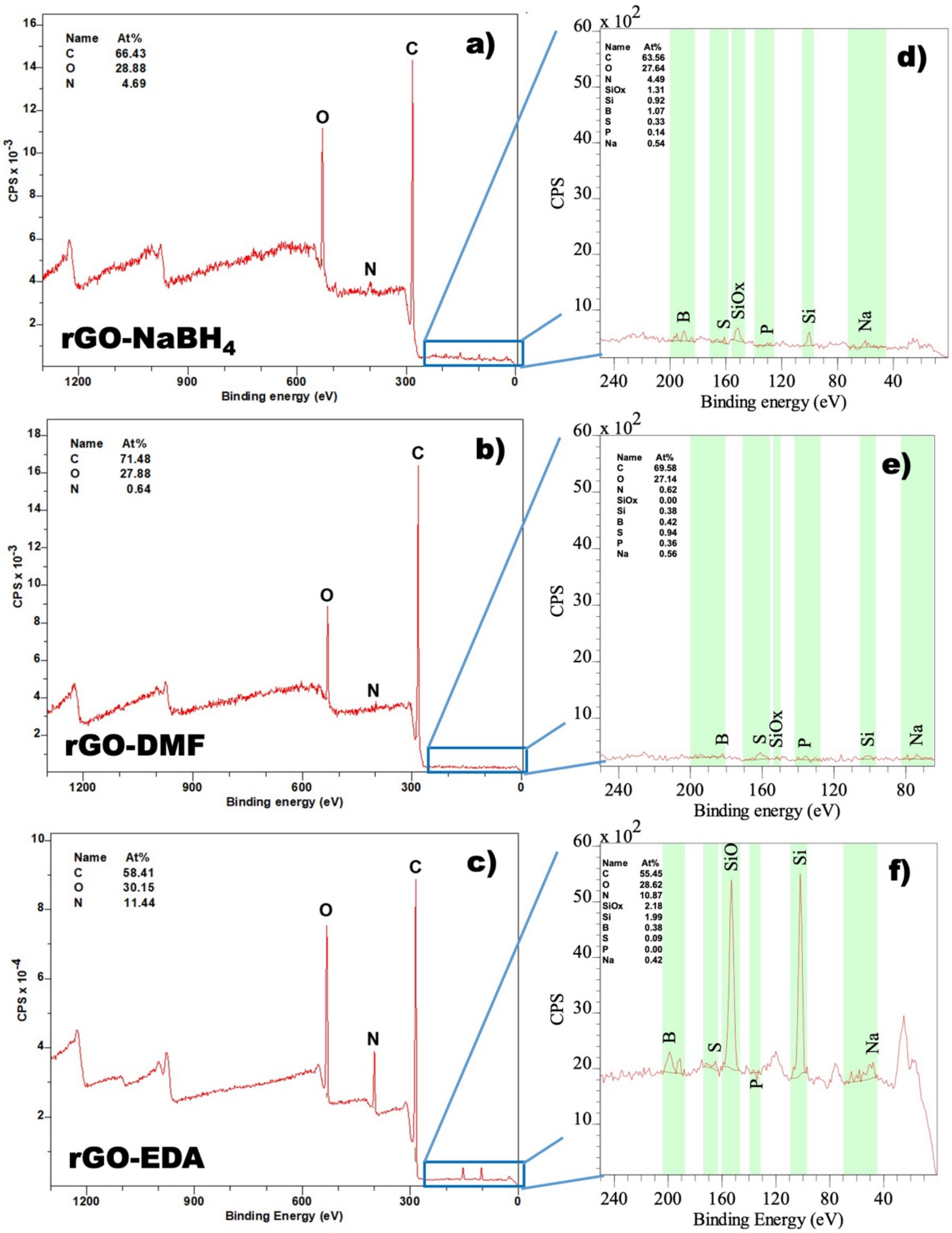

Figure S6. XPS survey spectra from the three different samples analyzed. (a)-(c) Present the carbon, nitrogen and oxygen peaks, as well as the atomic percentage quantification for each of the three samples. Notably, a considerably higher amount of nitrogen was measured when using EDA as the reducing agent. (d)-(f) show magnified views of the low binding energy values to account for the purity of the obtained samples (small traces present mainly as byproducts from the glassware).

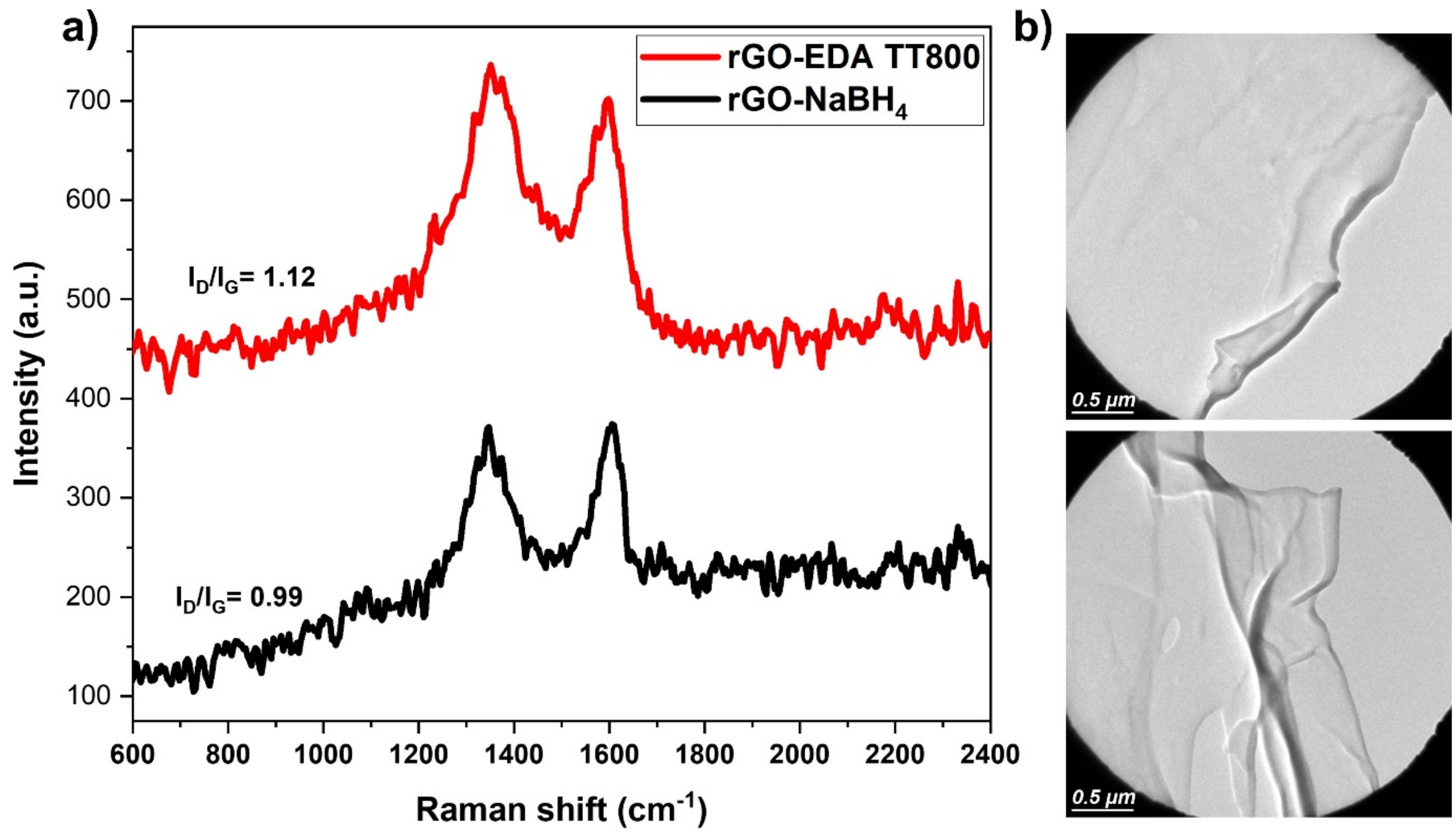

Figure S7. (a) Raman spectra of rGO-$NaBH_4$ and rGO-EDATT800 showing the characteristic D and G bands and their corresponding I_D/I_G ratios. (b) Representative TEM micrographs of rGO-EDATT800 revealing the typical sheet-like morphology of graphene-based materials.

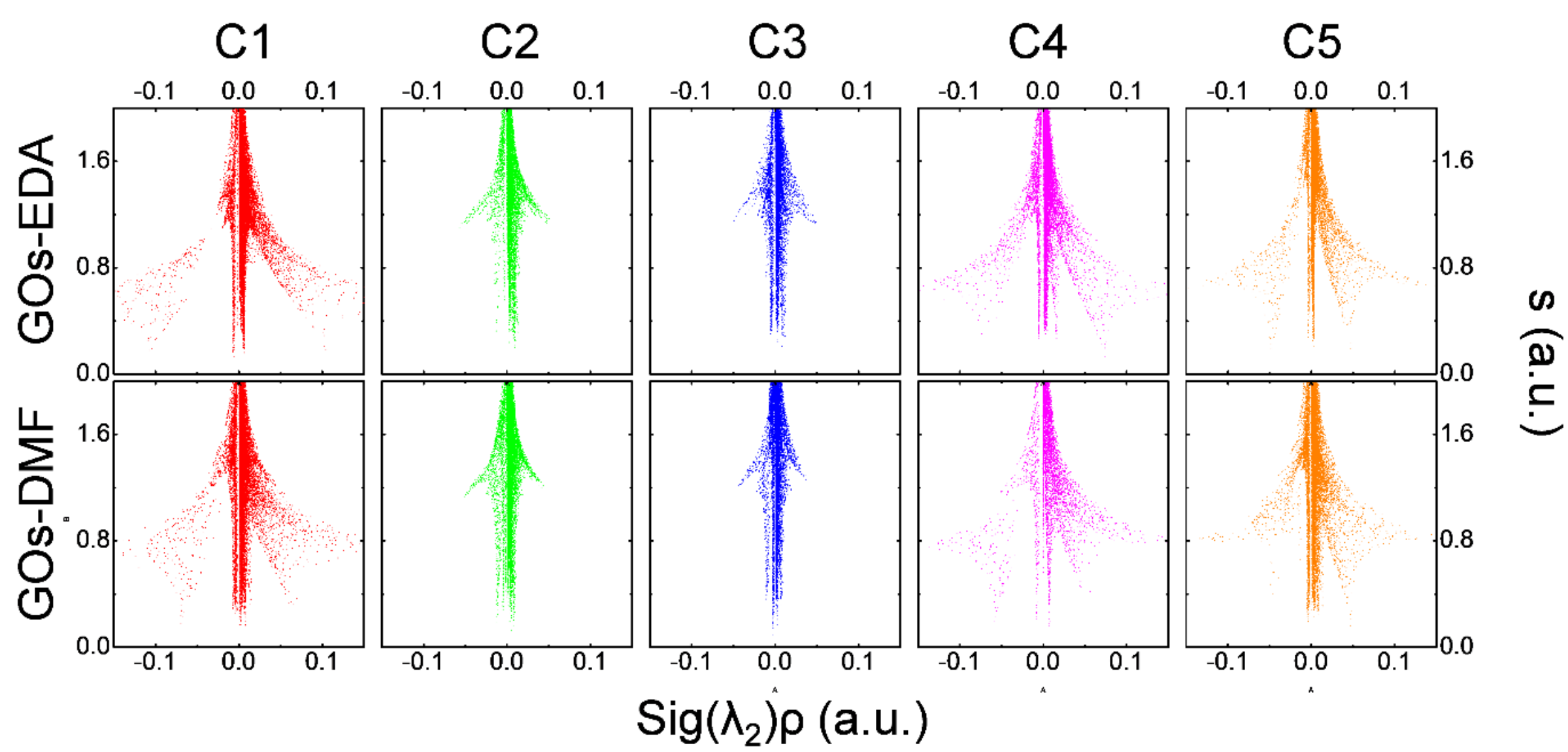

Figure S8. sig(λ_2)ρ graphs

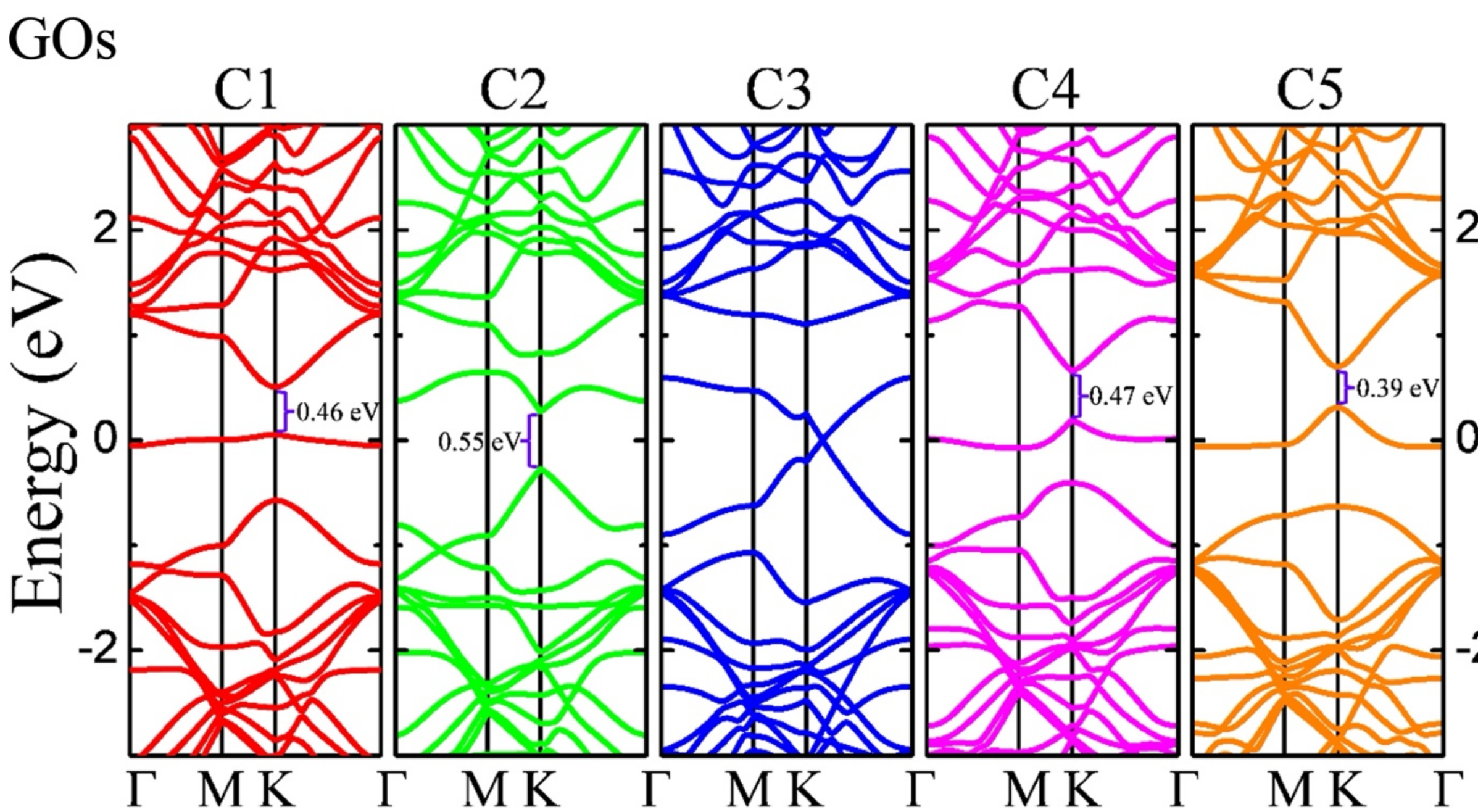

Figure S9. Band structure along the Γ-M-K-Γ pathway of the five different models of GOs considered in this work (see **Figure 3** in the main text).

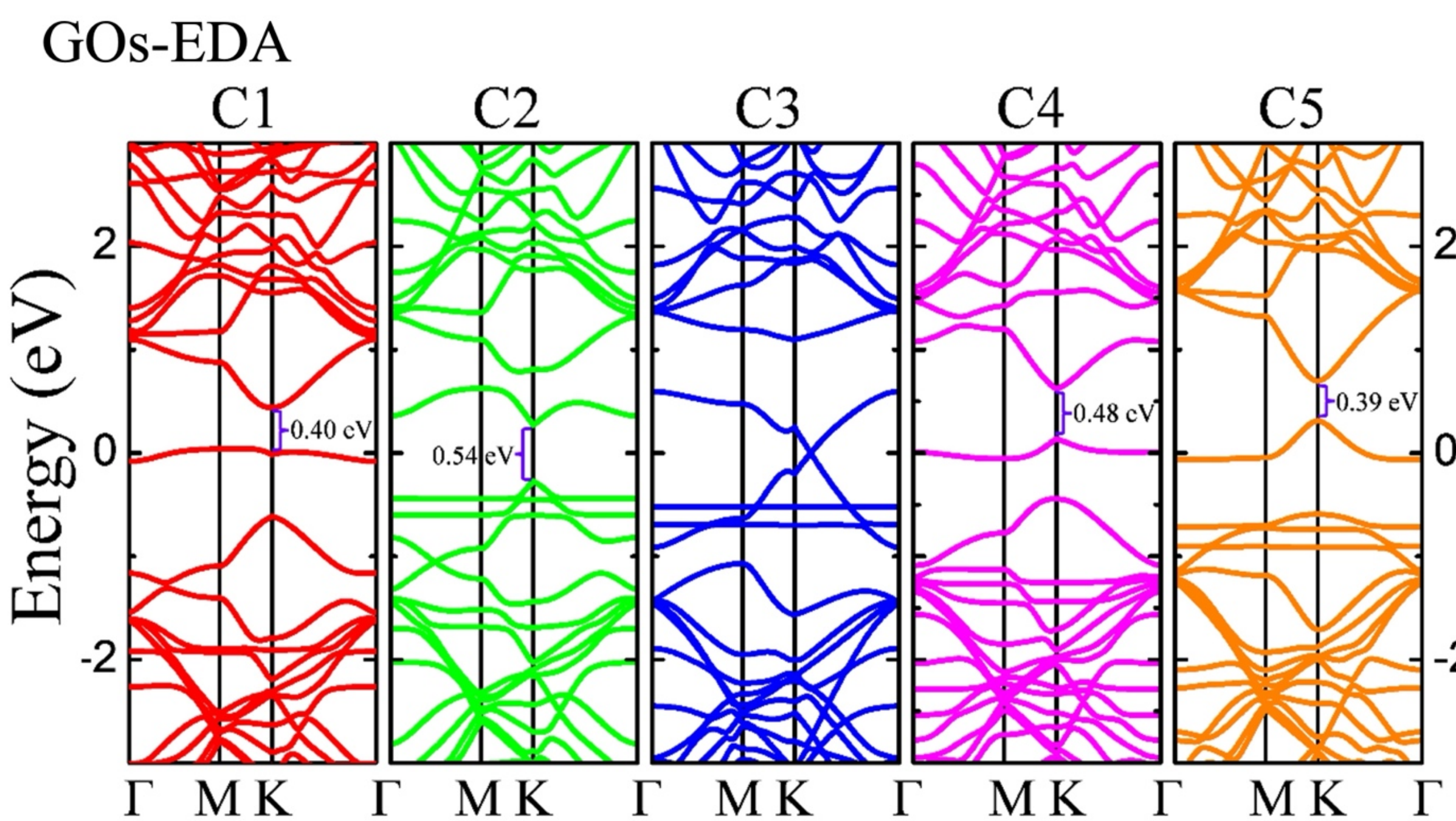

Figure S10. Band structure along the Γ-M-K-Γ pathway of the five different models of GOs when considering the adsorption of an **EDA** molecule.

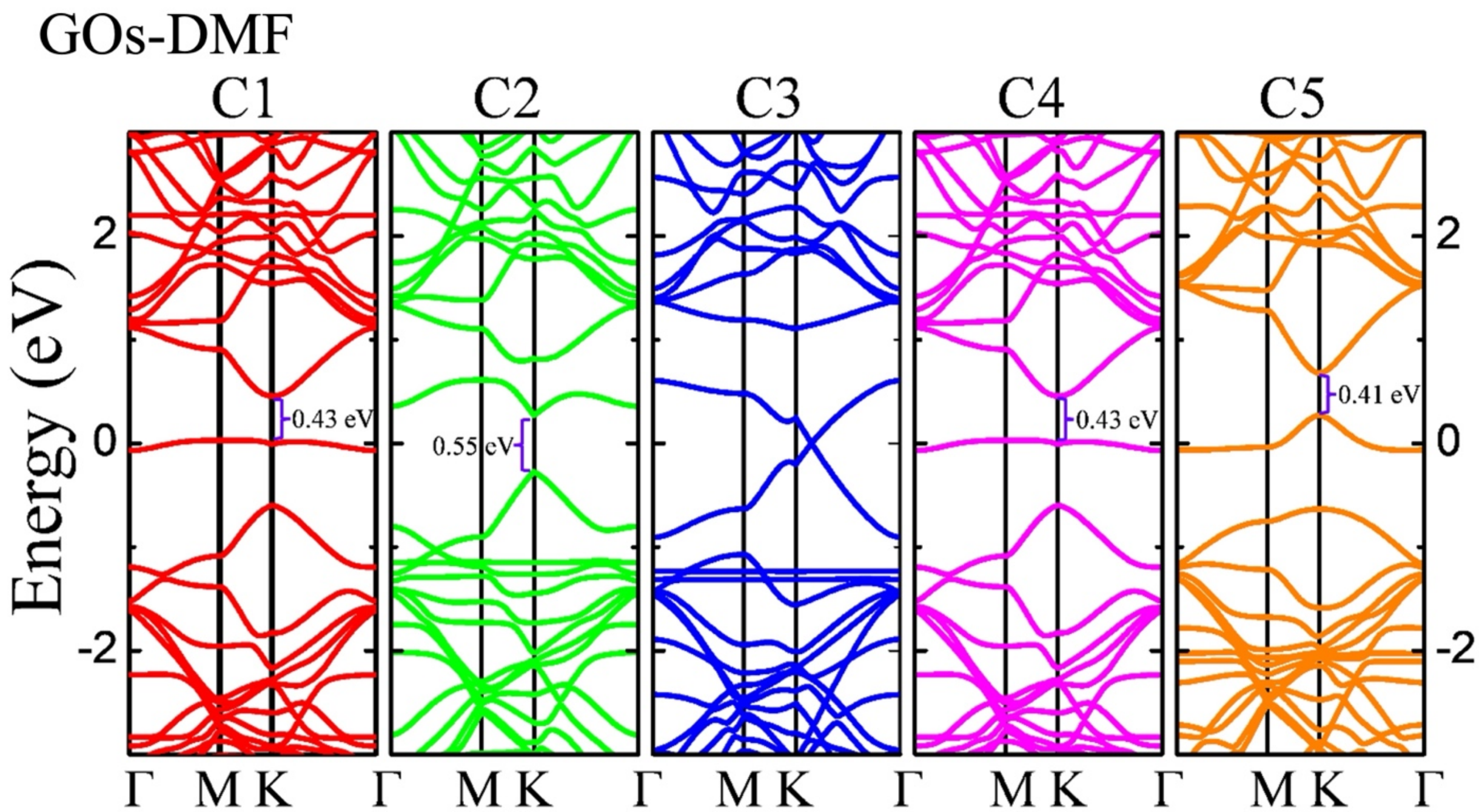

Figure 11. Band structure along the Γ-M-K-Γ pathway of the five different models of GOs when considering the adsorption of a **DMF** molecule.

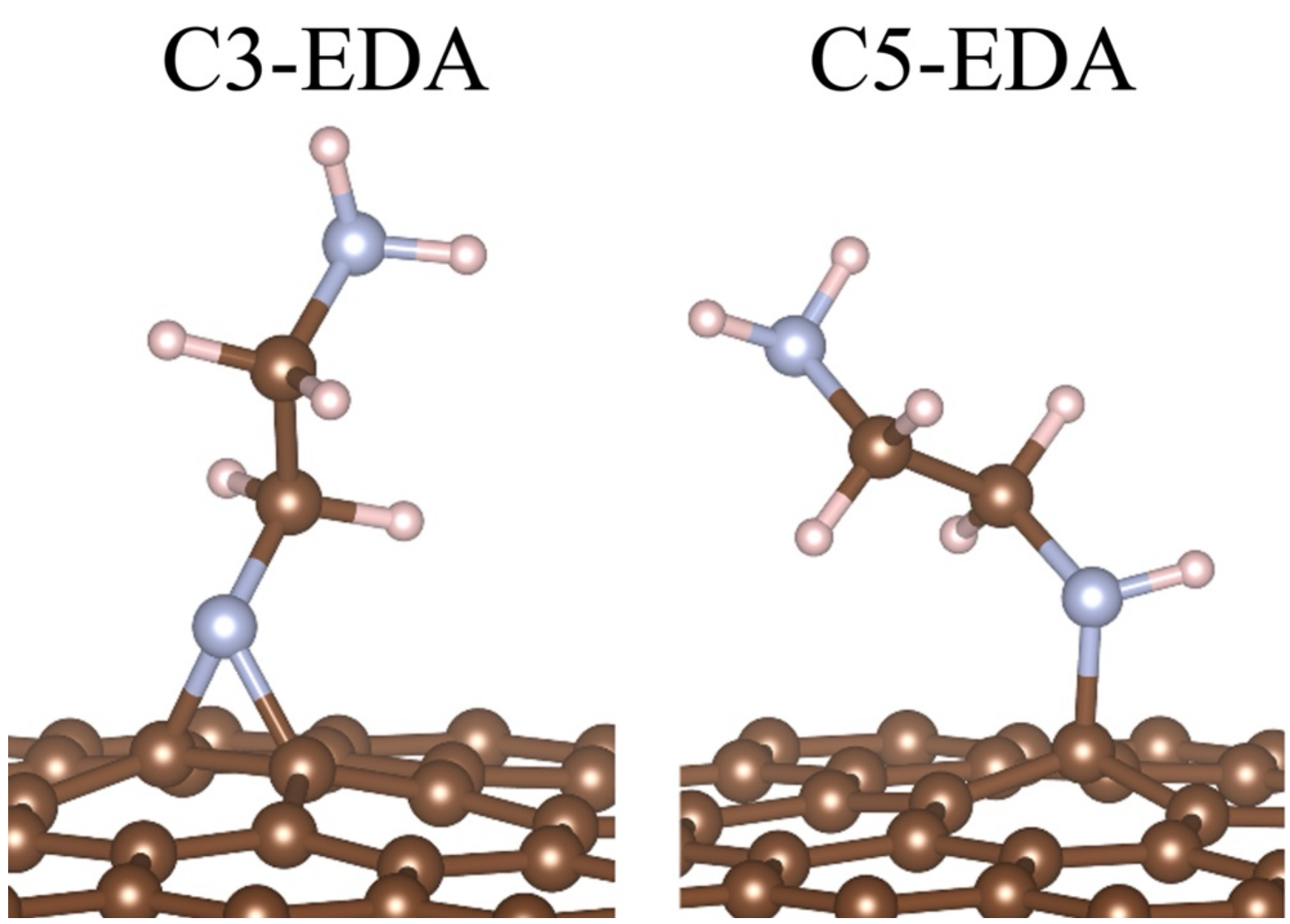

Figure S12. Relaxed structural models for the chemisorbed EDA molecule onto GOs corresponding to model C3 (epoxy group) and C5 (hydroxyl planar group) generating a tertiary amine or a secondary amine respectively.

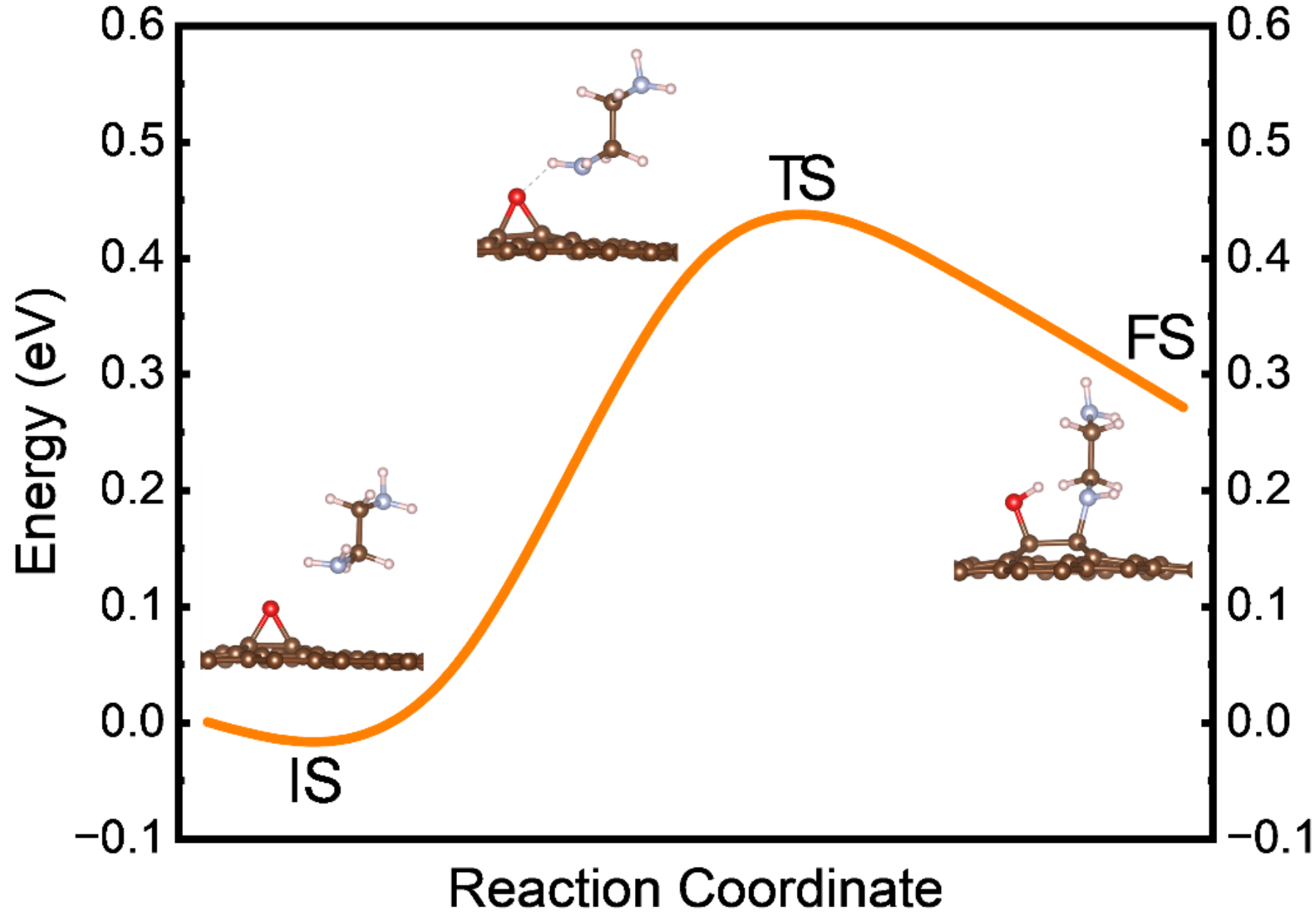

Figure S13. Minimum energy pathway for the covalent bond formation between EDA and GOs.

Table S2. FTIR peak assignment of N-rGOs samples after the 800°C thermal treatment.

**After Thermal Treatment**	
Wavenumber (cm^{-1})	Band Assigment
1095	C-O
1387	O-H
1458	C-H
1630	C=O
1646	C-N

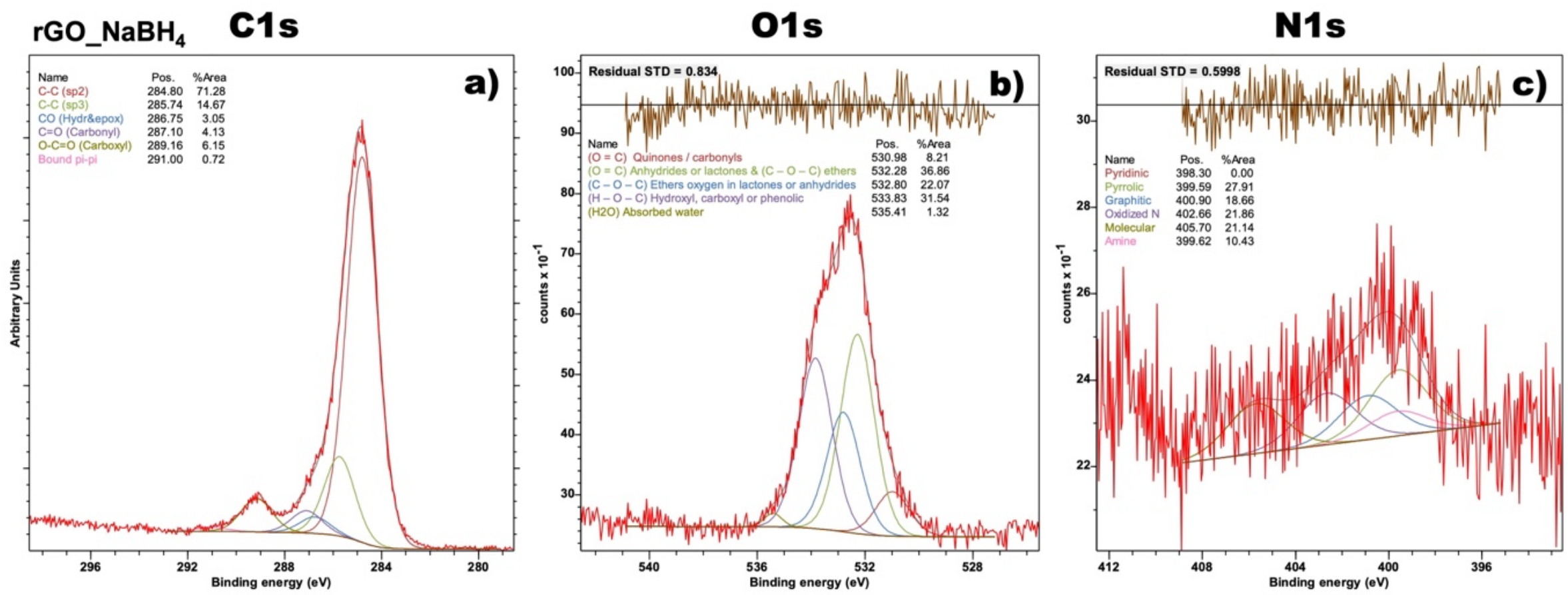

Figure S14. Deconvolution of the XPS spectra corresponding to the a) C1s data, b) O1s data and c) N1s data for the **rGO-NaBH₄_TT800°C** sample.

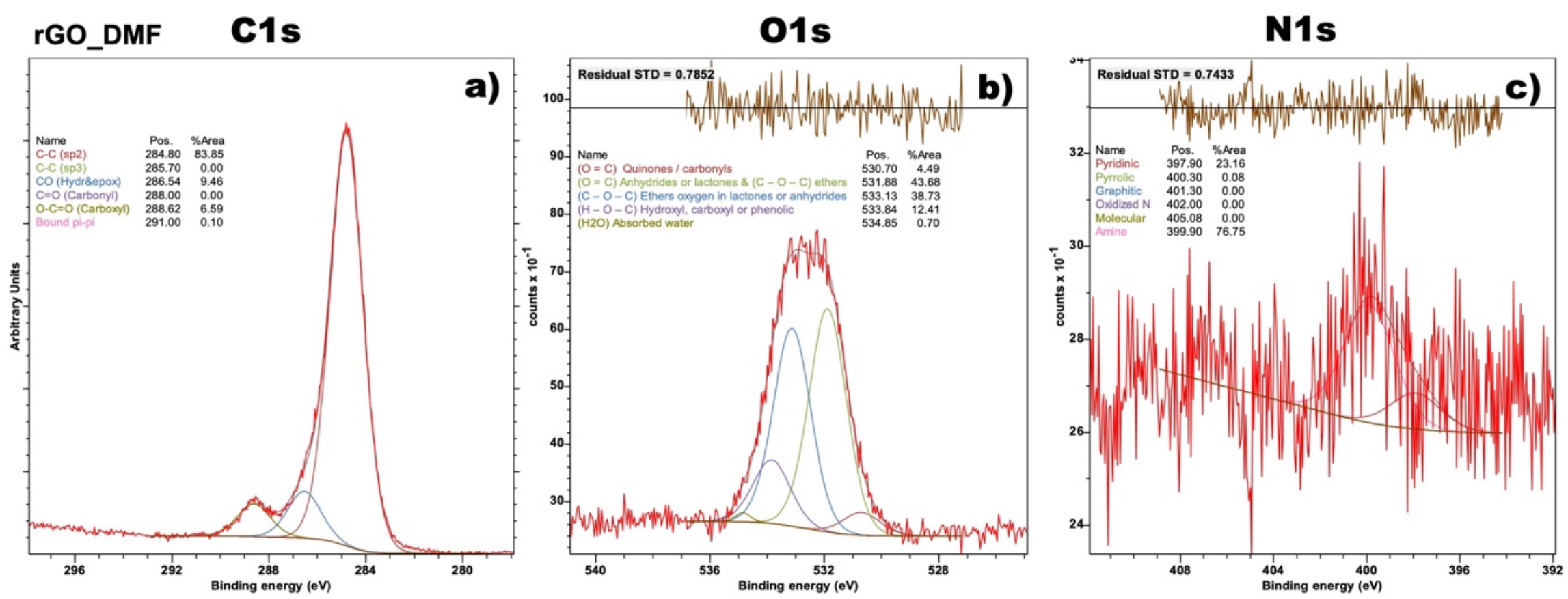

Figure S15. Deconvolution of the XPS spectra corresponding to the a) C1s data, b) O1s data and c) N1s data for the **rGO-DMF_TT800°C** sample.

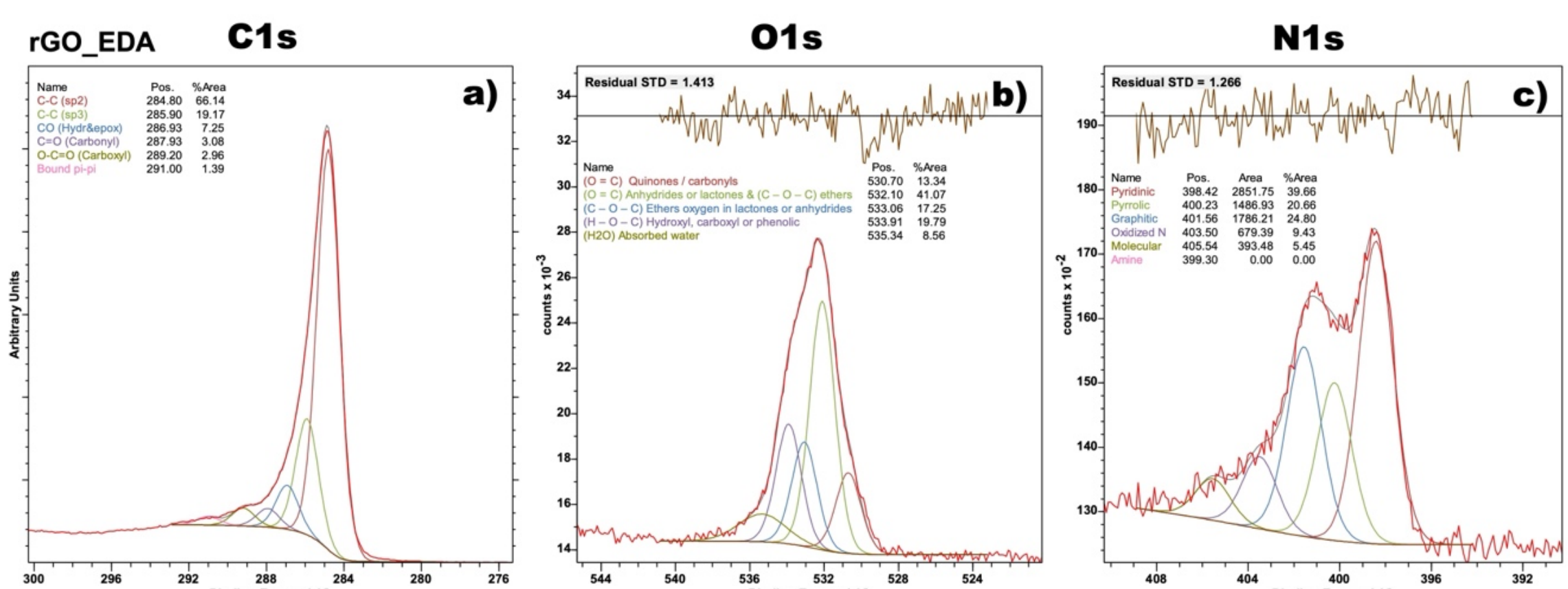

Figure S16. Deconvolution of the XPS spectra corresponding to the a) C1s data, b) O1s data and c) N1s data for the **rGO-EDA_TT800°C** sample.

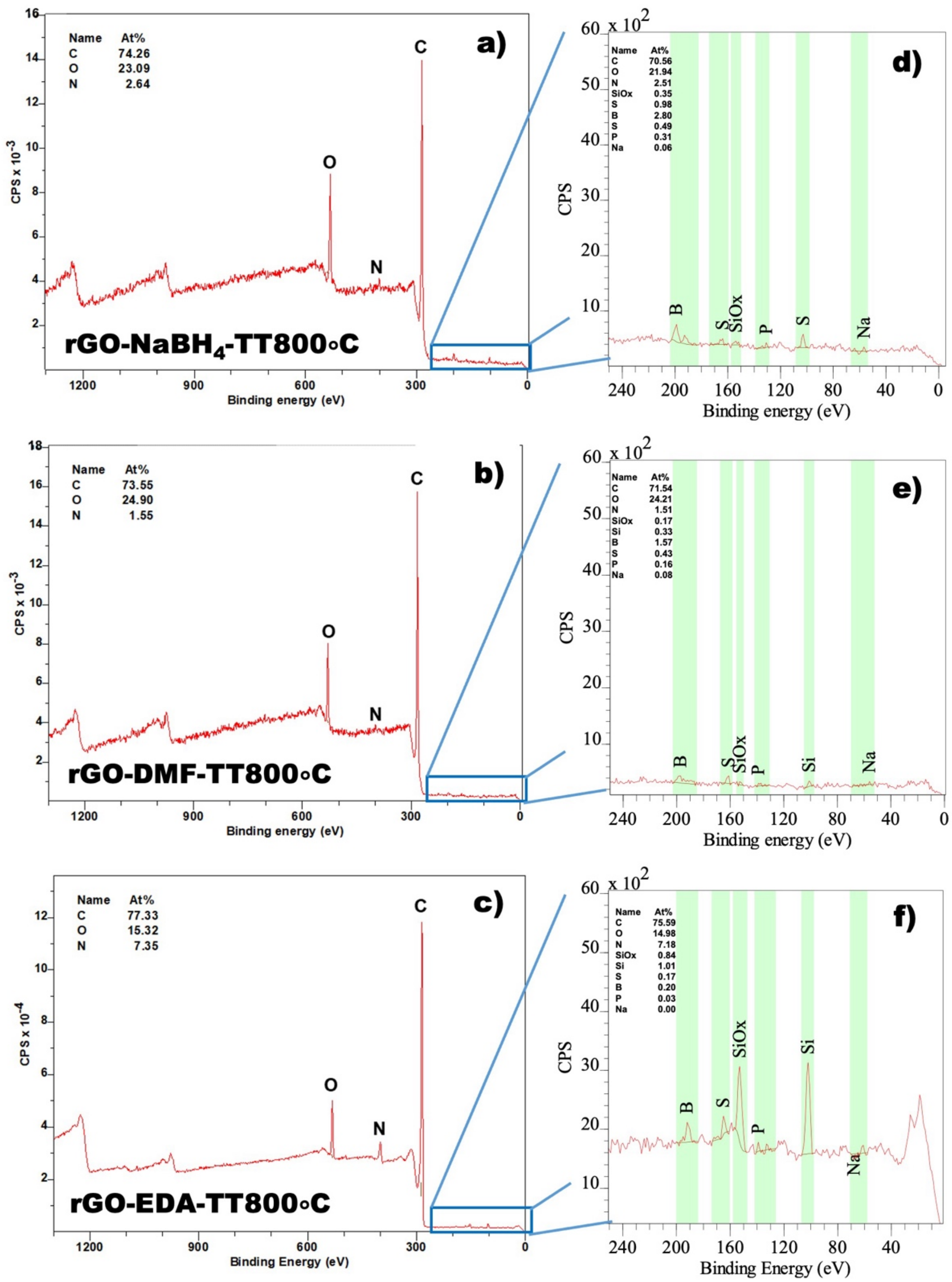

Figure S17. XPS survey spectra from the three different samples analyzed after the 800°C thermal treatment. (a)-(c) Present the carbon, nitrogen and oxygen peaks, as well as the atomic percentage quantification for each of the three samples. Notably, a considerably amount of nitrogen still present in the rGO-EDA-TT800°C after the thermal treatment. (d)-(f) show magnified views of the low binding energy values to account for the purity of the obtained samples (small traces present mainly as byproducts from the glassware).

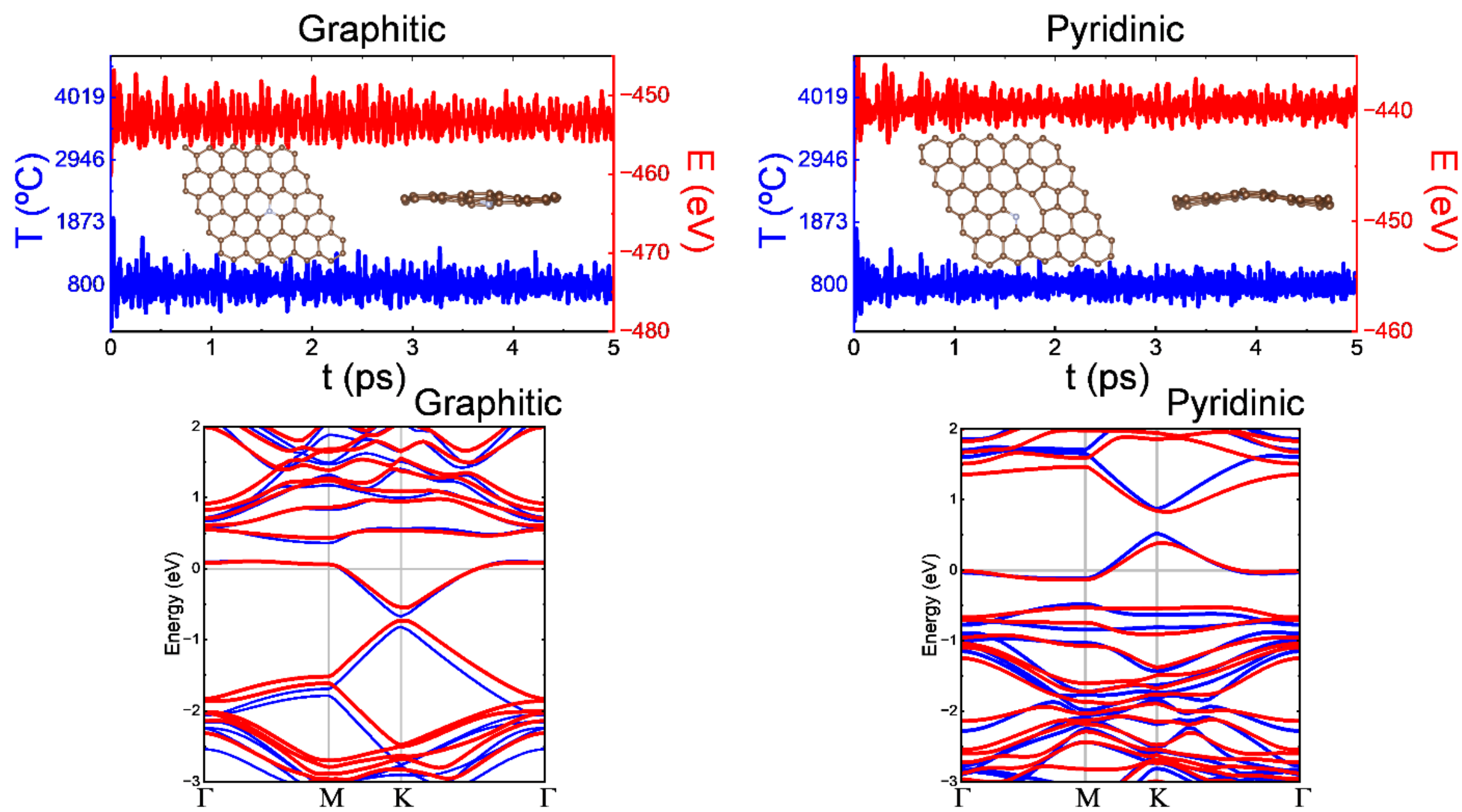

Figure S18. AIMD for graphitic and pyridinic N-doped graphene. Also, band structure along the Γ-M-K-Γ pathway, blue lines are for band structure of ideal models, while red lines are for models after AIMD simulation at 800 ºC.

Table S3. The peak current measured for the three rGO samples after the 800°C heat treatment from **Figure 7e** and after the non-faradaic current subtraction from **Figure 7f**.

**Sample**	**Peak current [mA/cm^2]**	**Peak current [mA/cm^2]** ***(after non-faradaic current subtraction)***
rGO-$NaBH_4$_TT800°C	-0.78	-0.72
rGO-EDA_TT800°C	-2.70	-2.78
rGO-DMF_TT800°C	-1.60	-1.64

Table S4. Onset potential data for the ORR previously reported.

Sample	Onset Potential	Technique	Conditions	Reference
N-Graphene (GRA N-max)	~ **0.83V vs RHE**	RRDE	O_2 saturated 0.1M KOH 1600rpm 10 mV/s	S[1]
N-rGO (G N-max)	~ **0.80V vs RHE**	RRDE	O_2 saturated 0.1M KOH 1600rpm 10 mV/s	S[1]
N-rGO (900°C)	~ **0.11V vs SHE** ~ -0.88V vs RHE	RDE	O_2 saturated 0.1M KOH 1600rpm 5 mV/s	S[2]
N-rGO (1000°C)	~ **0.08V vs SHE** ~ -0.85V vs RHE	RDE	O_2 saturated 0.1M KOH 1600rpm 5 mV/s	S[2]
N-rGO (PANi/RG-O)	~ **-0.12V vs Ag/AgCl** ~ -0.85V vs RHE	RDE	O_2 saturated 0.1M KOH 2500rpm 10 mV/s	S[3]
N-rGO (Ppy/RG-O)	~ **-0.11V vs Ag/AgCl** ~ -0.86V vs RHE	RDE	O_2 saturated 0.1M KOH 2500rpm 10 mV/s	S[3]
N-rGO (3-NG)	~ **-0.16V vs SCE** ~ -0.85V vs RHE	RDE	O_2 saturated 0.1M KOH 1900rpm 10 mV/s	S[4]
N-doped carbon nanohorns (g-N-CNH)	~ **0.68V vs RHE**	CV	O_2 saturated 0.1M NaOH 50 mV/s	S[5]
N-rGO (700°C-30min+700°C-2h)	~ **0.82V vs RHE**	RDE	O_2 saturated 0.1M KOH 1600rpm 20 mV/s	S[6]
N-rGO (900°C-30min)	~ **0.79V vs RHE**	RDE	O_2 saturated 0.1M KOH 1600rpm 20 mV/s	S[6]
N-rGO (900°C-30min)	~ **0.75V vs RHE**	RDE	O_2 saturated 0.1M KOH 1600rpm 20 mV/s	**This work**

References

(1) Cardoso, E. S. F.; Fortunato, G. V.; Palm, I.; Kibena-Põldsepp, E.; Greco, A. S.; Júnior, J. L. R.; Kikas, A.; Merisalu, M.; Kisand, V.; Sammelselg, V.; Tammeveski, K.; Maia, G. Effects of N and O Groups for Oxygen Reduction Reaction on One- and Two-Dimensional Carbonaceous Materials. *Electrochim. Acta* **2020**, *344*. https://doi.org/10.1016/j.electacta.2020.136052.

(2) Geng, D.; Chen, Y.; Chen, Y.; Li, Y.; Li, R.; Sun, X.; Ye, S.; Knights, S. High Oxygen-Reduction Activity and Durability of Nitrogen-Doped Graphene. *Energy Environ. Sci.* **2011**, *4* (3), 760–764. https://doi.org/10.1039/c0ee00326c.

(3) Lai, L.; Potts, J. R.; Zhan, D.; Wang, L.; Poh, C. K.; Tang, C.; Gong, H.; Shen, Z.; Lin, J.; Ruoff, R. S. Exploration of the Active Center Structure of Nitrogen-Doped Graphene-Based Catalysts for Oxygen Reduction Reaction. *Energy Environ. Sci.* **2012**, *5* (7), 7936–7942. https://doi.org/10.1039/c2ee21802j.

(4) Vikkisk, M.; Kruusenberg, I.; Joost, U.; Shulga, E.; Kink, I.; Tammeveski, K. Electrocatalytic Oxygen Reduction on Nitrogen-Doped Graphene in Alkaline Media. *Appl. Catal. B* **2014**, *147*, 369–376. https://doi.org/10.1016/j.apcatb.2013.09.011.

(5) Iglesias, D.; Giuliani, A.; Melchionna, M.; Marchesan, S.; Criado, A.; Nasi, L.; Bevilacqua, M.; Tavagnacco, C.; Vizza, F.; Prato, M.; Fornasiero, P. N-Doped Graphitized Carbon Nanohorns as a Forefront Electrocatalyst in Highly Selective O2 Reduction to H2O2. *Chem* **2018**, *4* (1), 106–123. https://doi.org/10.1016/j.chempr.2017.10.013.

(6) Ruiz-Marizcal, J. M.; Paez-Ornelas, J. I.; Fernández-Escamilla, H. N.; Murillo-Bracamontes, E. A.; Alonso-Núñez, G.; Perez-Tijerina, E. G.; Takeuchi, N.; Romo-Herrera, J. M. From Graphene Oxide to N-Doped Graphene: Understanding the Doping Process. *Advanced Energy and Sustainability Research* **2025**, *6* (4). https://doi.org/10.1002/aesr.202400310.
